# Eudesmane and Eremophilane Sesquiterpenes from the Fruits of *Alpinia oxyphylla* with Protective Effects against Oxidative Stress in Adipose-Derived Mesenchymal Stem Cells

**DOI:** 10.3390/molecules26061762

**Published:** 2021-03-21

**Authors:** Punam Thapa, Yoo Jin Lee, Tiep Tien Nguyen, Donglan Piao, Hwaryeong Lee, Sujin Han, Yeon Jin Lee, Ah-Reum Han, Hyukjae Choi, Jee-Heon Jeong, Joo-Won Nam, Eun Kyoung Seo

**Affiliations:** 1 College of Pharmacy, Yeungnam University, Gyeongsan, Gyeongsangbukdo 38541, Korea; pansup35@gmail.com (P.T.); tientiephup@gmail.com (T.T.N.); h5choi@yu.ac.kr (H.C.); jeeheon@yu.ac.kr (J.-H.J.); 2 College of Pharmacy, Graduate School of Pharmaceutical Sciences, Ewha Womans University, Seoul 03760, Korea; yoojin0909@hanmail.net (Y.J.L.); parkdl@ewhain.net (D.P.); ongsky119@naver.com (H.L.); sujinhan@ewhain.net (S.H.); lyjin94@naver.com (Y.J.L.); 3 Advanced Radiation Technology Institute, Korea Atomic Energy Research Institute, Jeollabuk-do, Jeongeup-si 56212, Korea; arhan@kaeri.re.kr

**Keywords:** *Alpinia oxyphylla*, eudesmane sesquiterpene, eremophilane sesquiterpene, adipose-derived mesenchymal stem cell, antioxidant, oxidative stress

## Abstract

*Alpinia oxyphylla* Miquel (Zingiberaceae) has been reported to show antioxidant, anti-inflammatory, and neuroprotective effects. In this study, two new eudesmane sesquiterpenes, 7α-hydroperoxy eudesma-3,11-diene-2-one (**1**) and 7β-hydroperoxy eudesma-3,11-diene-2-one (**2**), and a new eremophilane sesquiterpene, 3α-hydroxynootkatone (**3**), were isolated from the MeOH extract of dried fruits of *A. oxyphylla* along with eleven known sesquiterpenes (**4**–**14**). The structures were elucidated by the analysis of 1D/2D NMR, high-resolution electrospray ionization mass spectrometry (HRESIMS), and optical rotation data. Compounds (**1**–**3**, **5**–**14**) were evaluated for their protective effects against *tert*-butyl hydroperoxide (tBHP)-induced oxidative stress in adipose-derived mesenchymal stem cells (ADMSCs). As a result, treatment with isolated compounds, especially compounds **11** and **12,** effectively reverted the damage of tBHP on ADMSCs in a dose-dependent manner. In particular, **11** and **12** at 50 µM improved the viability of tBHP-toxified ADMSCs by 1.69 ± 0.05-fold and 1.61 ± 0.03-fold, respectively.

## 1. Introduction

**Figure 1:**
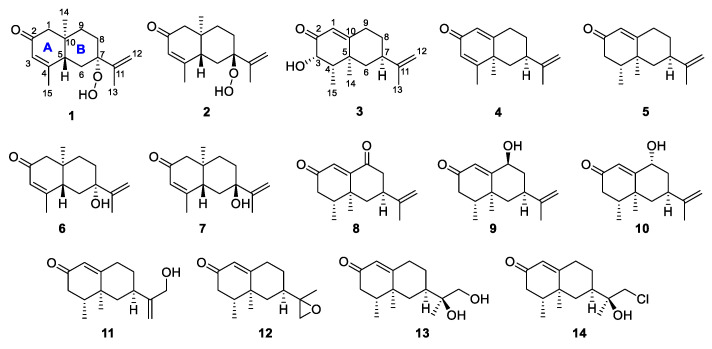
Chemical structures of compounds isolated from *Alpinia oxyphylla*.

*Alpinia oxyphylla* Miquel (Zingiberaceae) is a flowering plant, and the genus of *Alpinia* comprises 250 species worldwide and is mainly distributed across subtropical regions [1]. It has been used both as a food [2] and a traditional herbal medicine in Korea, China, and Japan [3,4]. The fruit of *A*. *oxyphylla* was reported to exhibit diverse pharmacological activities, such as anti-inflammatory [5], anti-ulcer [6], anti-allergy [7], and neuroprotective effects [8]. Previous studies have revealed the presence of different classes of chemicals in *A*. *oxyphylla*, including monoterpenes [9], sesquiterpenes [1,4,10], diterpenoids [11], flavonoids [12], diarylheptanoids [12], and steroids [13]. Among them, sesquiterpenes, such as 12-hydroxynootkatone [14], nootkatone [4], oxyphyllanene C, and oxyphyllanene E [1], are responsible for the inhibition of nitric oxide (NO) production in interferon-γ and lipopolysaccharide-treated RAW264.7 macrophage cells. As part of our ongoing project to investigate the antioxidant constituents of herbal medicines, the chemical exploration of *A*. *oxyphylla* led to the identification of three novel compounds: two eudesmane sesquiterpenes—7α-hydroperoxy eudesma-3,11-diene-2-one (**1**) and 7β-hydroperoxy eudesma-3,11-diene-2-one (**2**)—and an eremophilane sesquiterpene, 3α-hydroxynootkatone (**3**). The following known compounds were also identified as 3,4-dehydronootkatone (**4**) [15], nootkatone (**5**) [14], 7-*epi*-teucrenone (**6**) [16], teucrenone (**7**) [17], 11(12)-dien-2,9-dione (**8**) [18], 9β-hydroxynootkatone (**9**) [14], oxyphyllol B (**10**) [10], (4*R*, 5*S*, 7*R*)-13-hydroxynootkatone (**11**) [19], nootkatone-11,12-epoxide (**12**) [20], nootkatone-11,12-diol (**13**) [14], and (11*S*)-12-chloronootkaton-11-ol (**14**) by comparison with reported data [21] (Figure 1). The isolated compounds, except for **4**, due to the limited amount, were evaluated for their protective effects against *tert*-butyl hydroperoxide (tBHP)-induced oxidative stress in adipose-derived mesenchymal stem cells (ADMSCs). As a result, compounds **11** and **12** demonstrated potent cell-protective effects. tBHP is a powerful oxidizing compound that causes oxidative stress in stem cells. It results in reduced endogenous defense molecules, such as superoxide dismutase-SOD and glutathione. As a result, the cellular organelles such as mitochondria are damaged, which leads to cell death. In this study, we aimed to isolate sesquiterpenes from the dried fruits of *A. oxyphylla* and to preliminarily screen their effects of preventing the death of tBHP-toxified MSCs. Based on the results, the potent compounds will be chosen to further evaluate their effects on preserving the activity of intracellular defensive molecules in MSCs of interest in the next studies.

## 2. Results and Discussion

### 2.1. Structure Elucidation

**Figure 2:**
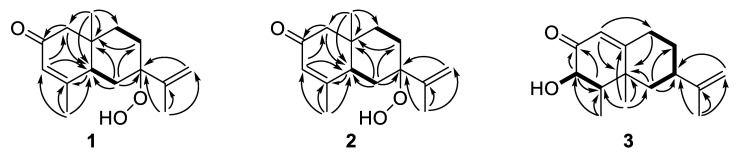
Key HMBC (H→C) and COSY (▬) correlations of **1–3.**

Compound **1** was isolated as a yellow oil. Its molecular formula, C_15_H_22_O_3_, with five degrees of unsaturation, was determined based on the high-resolution electrospray ionization mass spectrometry (HRESIMS) ion peak at *m*/*z* 251.1643 [M + H]^+^ (calculated for C_15_H_23_O_3_^+^, 251.1642). The ion peak at *m*/*z* 217.1598 [M + H − H_2_O_2_]^+^ [22,23] and neutral loss of 51 Da (H_2_O_2_ + NH_3_) from the ammonium adduct, *m*/*z* 268.1872 [M + NH_4_]^+^ [24] are characteristic evidence for the presence of the hydroperoxy group. The ^1^H-NMR spectrum of **1** showed three olefinic protons (*δ*_H_ 5.90 (dt, *J* = 2.9, 1.3 Hz, 1H), 5.37 (t, *J* = 1.3 Hz, 1H), 5.21 (brs, 1H)), one methine (*δ*_H_ 2.37 (m, 1H)), four methylenes (*δ*_H_ 2.41 (m, 1H)/1.54 (m, 1H), 2.30 (dd, *J* = 16.2, 1.3 Hz, 1H)/ 2.14 (dd, *J* = 16.2, 1.3 Hz, 1H), 2.19 (m, 1H)/1.78 (td, *J* = 14.1, 4.1 Hz, 1H), 1.53 (m, 1H)/1.42 (td, *J* = 13.9, 4.1 Hz, 1H), and three methyls (*δ*_H_ 0.96 (brs, 3H), 1.93 (t, *J* = 1.3 Hz, 3H), 1.84 (brs, 3H)). The ^13^C-NMR and distortionless enhancement by polarization transfer (DEPT) spectra displayed fifteen carbon signals, including one carbonyl group at *δ*_C_ 198.9; four quaternary carbons at *δ*_C_ 162.0, 141.5, 86.7, and 37.9; four methylenes at *δ*_C_ 54.1, 37.2, 28.7, and 26.7; an olefinic methylene at *δ*_C_ 118.5; three methyls at *δ*_C_ 22.1, 18.9 and 17.0; one methine at *δ*_C_ 44.5; and one olefinic methine at *δ*_C_ 127.2. The ^1^H and ^13^C-NMR spectra of **1** (Table 1 and Table 2) were found to be similar to those of the known eudesmane sesquiterpene, 7-*epi*-teucrenone (**6**) [16], except for a substitution of a tertiary hydroperoxy group (*δ*_C_ 85.9) [25,26] instead of a tertiary hydroxy group (*δ*_C_ 74.4) [16] at C-7, which was supported by the downfield shifted signal of C-7 (*δ*_C_ 86.7). Five degrees of unsaturation indicated a dicyclic ring system possesses one carbonyl group and two double bonds. The planar structure of **1** was established based on a comprehensive analysis of COSY, HSQC, and HMBC correlations, as shown in Figure 2. Key HMBC correlations from H-1 to C-2/C-5, H-3 to C-1/C-5/C-15, H-15 to C-3/C-4/C-5, and H-5 to C-15 were used to elucidate ring A. Ring B was confirmed by HMBC correlations from H-6 to C-5/C-7/C-8/C-10 and H-8 to C-10. The HMBC correlations from H-12 to C-7 and H-13 to C-7/C-11/C-12 suggested an isopropylidene group positioned at C-7. All figures of HRESIMS and 1D, 2D NMR of compound **1** were provided in supplementary material (Figures S1–S8).

**Figure 3:**
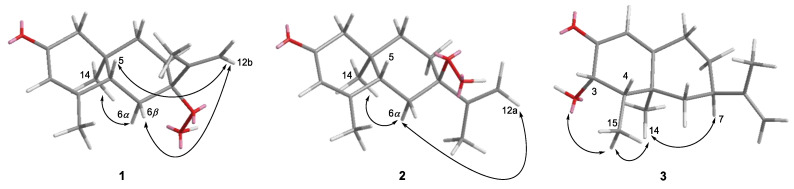
Key NOESY (H↔H) correlations of **1**–**3.**

The relative configuration of **1** was determined through the NOESY experiment (Figure 3). The NOE correlations between H-6β and H-12b as well as H-6α and H_3_-14 indicated that both the C-7 hydroperoxide and C-14 methyl groups were α*-*positioned. In addition, the NOE correlation between H-5 and H-12b suggested a β*-*oriented proton at C-5. The optical rotation was obtained to determine the absolute configuration of **1**; a weak negative specific rotation, ${\left[\alpha \right]}_{D}^{25}$: −1.2 (*c* 0.25, CH_3_OH), was observed, indicating the presence of a racemic mixture. Based on these observations, the structure of **1** was elucidated as 7α-hydroperoxy eudesma-3,11-diene-2-one.

Compound **2** was isolated as a white powder. Its molecular formula, C_15_H_22_O_3_, with five degrees of unsaturation, was confirmed based on the HRESIMS ion peak at *m*/*z* 251.1639 [M + H]^+^ (calcd for C_15_H_23_O_3_^+^, 251.1642) (Figure S16). The fragmentation pattern showed the same pattern as mentioned in **1** to confirm the presence of a hydroperoxy group. The comparison of the ^1^H and ^13^C-NMR spectroscopic data revealed that the planar structure of **2** was identical to that of **1**, which was further supported by the analysis of the 2D NMR spectra, as shown in Figure 2 (Figures S9–S15). The relative configurations of a hydroperoxy group at C-7 in the eudesmane sesquiterpene were determined using ^13^C-NMR chemical shift differences at C-11 and C-12 [16,17] as well as NOESY spectrum. The resonances for C-11 and C-12 in **2** were downfield and upfield shifted, respectively, both by 6 ppm, compared to those of **1**, suggesting that the relative configurations at C-7 in **2** were different from those in **1**. The H-6α was observed as dd with relatively large coupling constants of 14.0 Hz (^2^*J*_H-6α, H-6β_) and 13.2 Hz (^3^*J*_H-5, H-6α_), which indicated H-6α and H-6β are axial and equatorial orientations, respectively. The NOE correlations between H-6α and H-14, as well as between H-6α and H-12a revealed that an isopropylidene group at C-7 of **2** was positioned on the same side with a methyl group (H_3_-14). Again, to determine the absolute configuration of **2**, the optical rotation was obtained, and a weak positive specific rotation, ${\left[\alpha \right]}_{D}^{25}$: +3.16 (*c* 0.19, CH_3_OH), was observed, concluding the presence of a racemic mixture. Based on these observations, the structure of **2** was elucidated as 7β-hydroperoxy eudesma-3,11-diene-2-one, a 7-epimer of **1**.

Compound **3** was isolated as a white powder. Its molecular formula, C_15_H_22_O_2_, with five degrees of unsaturation, was determined based on the HRESIMS ion peak at *m*/*z* 235.1692 [M + H]^+^ (calcd for C_15_H_23_O_2_^+^, 235.1693) (Figure S24). The ^1^H-NMR spectroscopic data (Table 1) indicated three olefinic protons (*δ*_H_ 5.84 (d, *J* = 1.5 Hz, 1H), 4.75 (t, *J* = 1.5 Hz, 1H), 4.72 (brs, 1H)), three methyls (*δ*_H_ 1.72 (brs, 3H), 1.28 (brs, 3H), 0.92 (d, *J* = 7.1 Hz, 3H)), three methylenes (*δ*_H_ 1.81 (t, *J* = 12.3 Hz, 1H)/1.76 (m, 1H), 2.07 (m, 1H)/1.32 (dd, 13.1, 4.4 Hz, 1H), 2.55 (tdd, *J* = 12.9, 4.5, 1.5 Hz, 1H)/2.29 (tdd, *J* = 12.9, 4.5, 2.7 Hz, 1H)), and three methines (including oxygenated methine) at *δ*_H_ 4.39 (dd, *J* = 5.3, 2.2 Hz, 1H), 2.59 (m, 1H) and 2.03 (dq, *J* = 6.1, 5.3 Hz, 1H). The ^13^C-NMR spectrum showed fifteen carbon signals: three methyls (*δ*_C_ 22.7, 21.1, and 9.0), three methylenes (*δ*_C_ 43.7, 34.6, and 33.1), one olefinic methylene *δ*_C_ 109.9, three methines (*δ*_C_ 73.4, 44.0, and 40.1), one olefinic methine (*δ*_C_ 119.6), three quaternary carbons (*δ*_C_ 170.7, 148.3, and 41.3), and one carbonyl (*δ*_C_ 199.8). Based on the ^1^H and ^13^C-NMR spectral analyses of **3** (Figures S17 and S18, Table 1), the structure was similar to that of an eremophilane sesquiterpene, nootkatone (**5**), except for the presence of an additional hydroxy group at C-3 in **3** [27]. Analysis of the COSY, HSQC, and HMBC spectroscopic data (Figures S20–S22) revealed the connectivity of the structure. The key HMBC correlations were as follows: from H-1 to C-5/C-9, H-3 to C-2/C-4, H-15 to C-3/C-4, H-4 to C-2, H-14 to C-4/C-5/C-6/C-10, H-6 to C-5/C-7/C-8, and H-9 to C-5, confirming the core planar structure of **3**. An isopropylidene group at C-7 was confirmed based on the HMBC correlations from H-12 to C-7 and H-13 to C-7/C-11/C-12. The relative configuration of **3** was assigned by NOE correlations, as shown in Figure 3 (Figure S23). The NOE correlations between H-7/H_3_-14, H_3_-14/H_3_-15, and H_3_-15/OH-3 suggested the α*-*orientation of the H-7, H_3_-14, H_3_-15, and β-orientation of H-3, which is further supported by the absence of NOE correlation between OH-3 and H-4. The absolute configuration of **3** was determined as 3*S*, 4*S*, 5*S*, 7*R* by comparing the optical rotation value, ${\left[\alpha \right]}_{D}^{25}$: +6.18 (*c* 0.15, CH_3_OH), with that of (4*R*,5*S*,7*R*)-nootkatone (**5**) [27]. Accordingly, the structure of **3** was elucidated as (3*S*,4*S*,5*S*,7*R*)-3α-hydroxynootkatone.

### 2.2. Biological Activities of Isolated Compounds on ADMSCs

**Figure 4:**
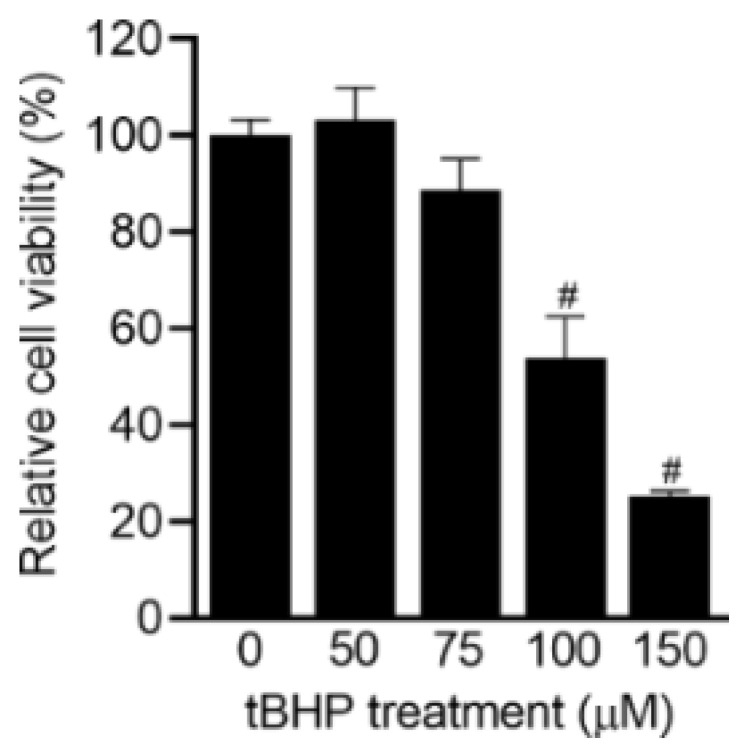
Effect of *tert*-butyl hydroperoxide (tBHP) on the viability of ADMSCs, evaluated by a CCK-8 assay (*n* = 5). The cell viability test was performed 24 h after treatment. # *p* < 0.0001.

**Figure 5:**
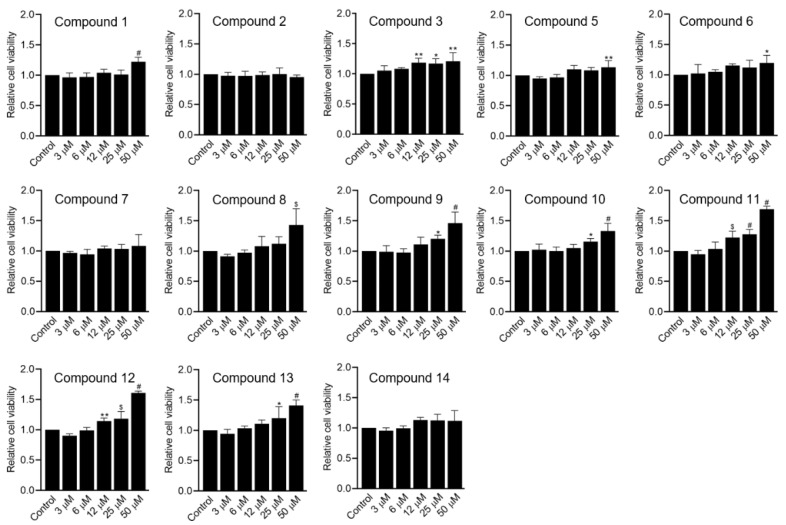
Effect of the isolated compounds on the viability of tBHP-damaged ADMSCs, evaluated by a CCK-8 assay (*n* = 5). ADMSCs were treated with a medium containing 100 µM tBHP and the isolated compounds at various concentrations. Cells only treated with 100 µM tBHP were used as controls. The cell viability test was performed at 24 h after treatment. The data represent two independent experiments. * *p* < 0.05, ** *p* < 0.01, $ *p* < 0.001, # *p* < 0.0001.

In this study, we tested the protective effects of compounds **1**–**3** and **5**–**14** on oxidative stress-induced ADMSCs. The activity of compound **4** could not be evaluated due to amount limitations. *Tert*-butyl hydroperoxide (tBHP) was used as an inducer of ADMSC death by generating excessive intracellular reactive oxygen species and reducing the activity of endogenous defensive molecules, such as superoxide dismutase-SOD and glutathione [28]. It was found that tBHP triggered ADMSC death dose-dependently (Figure 4). Particularly, the relative viability of ADMSCs after exposure to 0, 50, 75, 100, and 150 µM tBHP was 100.0 ± 3.3%, 103.3 ± 6.6%, 88.7 ± 6.4%, 53.8 ± 8.6% (*p* < 0.0001), and 25.1 ± 1.3% (*p* < 0.0001), respectively. To investigate the effects of the isolated compounds, 100 µM tBHP was chosen to induce oxidative stress in ADMSCs. Figure 5 shows the relative cell viability of the control (only tBHP treatment) and treated groups (co-treatment with tBHP and compounds). Interestingly, compounds **8** to **13** effectively prevented oxidative stress-induced ADMSC death, as indicated by a minimum 1.4-fold improvement in cell viability at 50 µM. Among these, compounds **11** and **12** exhibited the highest protective effects, which were improved by 1.69 ± 0.05-fold (*p* < 0.0001) and 1.61 ± 0.03-fold (*p* < 0.0001), respectively, at 50 µM. Meanwhile, compounds **3** and **6** only slightly inhibited the toxic effects of tBHP on ADMSCs, with 1.21 ± 0.14-fold and 1.20 ± 0.12-fold higher cell viability at 50 µM, respectively. We found that all the remaining compounds were ineffective in recovering ADMSCs from chemically induced oxidative stress at all tested concentrations. Further analysis would be necessary to investigate the relationship between structure and its effect.

*A. oxyphylla* is commonly used for its antioxidant, anti-inflammatory, and neuroprotective effects, but the effects of its active compounds remain to be explored [29,30,31,32]. In the present study, we successfully proved the protective effects of 13 isolated sesquiterpenes on the viability of ADMSCs exposed to tBHP-induced oxidative stress. In neurodegenerative diseases, neuronal stem cells experience a high level of oxidative stress, which impairs their regenerative capacity [33]. Although we could not isolate neuronal stem cells for the test, our results provide clear evidence for the prevention of oxidative stress in ADMSCs using several potent isolated sesquiterpenes. Future studies would be required to investigate the impact of these compounds in in vivo neurodegenerative disease models.

## 3. Materials and Methods

### 3.1. General Procedures

Optical rotation was measured using a JASCO P-2000 polarimeter (Tokyo, Japan). The NMR spectra were acquired on a 400 MHz Agilent NMR spectrometer (DD2, Santa Clara, CA, USA) using CDCl_3_. The HRESIMS was performed on an Agilent 6220 Accurate-Mass TOF LC/MS system. Silica gel (230–400 mesh, Merck KGaA, 64271 Darmstadt, Germany) and RP-18 (YMC gel ODS-A, 12 nm, S-150 μm, YMC Co., Ltd., Kyoto 600-8106, Japan). Thin Layer Chromatography (TLC) analysis was performed on silica gel 60 F254 (0.2 mm thickness, Merck KGaA, 64271 Darmstadt, Germany) and RP-18 F 254_S_ (Merck KGaA, 64271 Darmstadt, Germany) plates by visualization under UV light at 254 nm, 365 nm, and 10% (*v/v*) of sulfuric acid followed by heating. Preparative HPLC was performed using an Acme 9000 system (Young Lin, Anyang, Korea) equipped with a YMC-Pack Pro C18 column (5 μm, 250 × 20 mm i.d.).

### 3.2. Plant Materials

The dried fruits of *A. oxyphylla* were purchased from the Insan Oriental Herbal Market in Seoul, Korea. A voucher specimen (No. EA323) was deposited at the Natural Product Chemistry Laboratory at Ewha Womans University, Seoul, Korea.

### 3.3. Extraction and Isolation

The dried fruit of *A. oxyphylla* (3 kg) was extracted with 100% MeOH three times for 48h at room temperature. The resultant extract after removing solvent was dissolved in H_2_O then fractionated with hexanes, methylene chloride (MC), EtOAc, and *n*-BuOH, sequentially. The hexane-soluble fraction (94 g) was subjected to a silica gel column using hexanes:EtOAc (99:1 to 50:50) as a step of the gradient solvent system to yield 19 subfractions (Fr. A1–A19). Subfraction Fr. A8 (13.9 g) was chromatographed over a silica gel column and eluted with hexanes:acetone (99:1 to 98:2) to afford 17 fractions (Fr. B1–B17). Reversed-phase (RP) C_18_ column chromatography (CC) was carried out with Fr. B7 (5.3 g) using MeOH:H_2_O (2:1 to 3:1) to yield 15 subfractions (Fr. C1–C15). Subfraction Fr. C9 (2.97 g) was chromatographed over a silica gel column eluted with hexanes:EtOAc (99:1 to 0:100) to obtain 25 subfractions (Fr. D1–D25). The combined subfraction Fr. D7–D9 (1.40 g) was fractionated over a silica gel column by elution with hexanes:EtOAc (98:2 to 90:10) to yield compound **5** (1.45 mg). Fr. D13–D15 (125.63 mg), eluted with hexanes:EtOAc (99:1 to 60:30), was chromatographed using a silica gel column to obtain 13 fractions (Fr. E1–E13). Fr. E6 (70.20 mg) was subjected to RP-C18 CC using MeOH:H_2_O (2:1) to give thirteen subfractions (Fr. F1-F13), and compound **3** (1.46 mg) was obtained from Fr. F7. Subfraction Fr. F8 (30.60 mg) was further fractionated using RP-C18 CC and eluted with MeOH:H_2_O (1:1 to 2:1) to isolate compound **8** (16.14 mg). The combined subfraction Fr. D16–D17 (110.20 mg) was subjected to a silica gel column by elution with hexanes:EtOAc (99:1 to 80:20) to afford 12 subfractions (Fr. G1–G12). The combined subfraction Fr. G8–G9 (22 mg) was chromatographed over an RP-C18 column and eluted with MeOH:H_2_O (1:1 to 2:1) to afford 10 fractions (Fr. H1–H10). Compound **13** (2.56 mg) was isolated after RP-C18 chromatography of the combined subfraction Fr. H4–H5 (10.20 mg) by elution with MeOH:H_2_O (1:1 to 2:1) to afford 8 fractions Fr. I1–I8. Subfraction Fr. I4 (1.29 mg) was purified using RP-C18 HPLC with MeOH:H_2_O (9:7) as the mobile phase to obtain compound **12** (1.25 mg). The combined subfraction Fr. G10–G11 (16.77 mg) was chromatographed over an RP-C18 open column using MeOH:H_2_O (1:1 to 2:1) to yield compounds **4** (0.94 mg) and **14** (0.80 mg). The combined subfraction D19–D20 (2.97 g) was chromatographed over an RP-C18 column by elution with MeOH:H_2_O (4:1 to 1:0) to obtain 11 subfractions (Fr. L1–L11). Along with these eleven fractions (Fr. L1–L11), **2** (19.04 mg) and **9** (20.22 mg) were purified using an RP-C18 column with MeOH:H_2_O (2:1 to 1:0) from the combined subfraction Fr. L3–L4 (1.54 g). Compound **1** (5.00 mg) was separated from subfraction Fr. M4 with RP-C18 HPLC using MeOH:H_2_O (2:1, 0.1% acetic acid in MeOH) as the mobile phase. Subfraction Fr. L5 (1.37 g) was fractionated with RP-C18 CC with MeOH:H_2_O (3:1) as the eluent to afford **10** (80.15 mg). Subfraction Fr. D21 (109.42 mg) was chromatographed over an RP-C18 column and eluted with MeOH:H_2_O (1:1 to 1:0) to afford 19 fractions (Fr. K1–K19), including compound **11** (12.74 mg). Subfraction Fr. K7 (13.02 mg) was subjected to RP-C18 CC and eluted with MeOH:H_2_O (2:1 to 1:0) to isolate compound **6** (7.14 mg). We separated compound **7** (0.90 mg) using RP-C18 HPLC with MeOH:H_2_O (3:1) as a solvent system from subfraction Fr. K13.

(5S*,7S*,10R*)-7α*-Hydroperoxy eudesma-3,11-diene-2-one (**1**):* yellow oil; ${\left[\alpha \right]}_{D}^{25}$: −1.2 (*c* 0.25, CH_3_OH); ^1^H and ^13^C-NMR, see Table 1 and Table 2; HRESIMS [M + H]^+^ *m*/*z* 251.1643 ([M + H]^+^ (calcd for C_15_H_23_O_3_^+^, 251.1642).

(5S*,7R*,10R*)-7β*-Hydroperoxy eudesma-3,11-diene-2-one (**2**):* white powder; ${\left[\alpha \right]}_{D}^{25}$: +3.16 (*c* 0.19, CH_3_OH); ^1^H and ^13^C-NMR, see Table 1 and Table 2; HRESIMS [M + H]^+^ *m*/*z* 251.1639 ([M + H]^+^ (calcd for C_15_H_23_O_3_^+^, 251.1642)

*(3S,4S,5S,7R)-3α-Hydroxynootkatone (**3**):* white powder; ${\left[\alpha \right]}_{D}^{25}$: +6.18 (*c* 0.15, CH_3_OH); ^1^H and ^13^C-NMR, see Table 1 and Table 2; HRESIMS [M + H]^+^ *m*/*z* 235.1692 (calcd for C_15_H_23_O_2_^+^, 235.1693).

### 3.4. Culture of Mouse ADMSCs

Experiments on animals were performed according to the national guidelines and with the approval of the Institutional Ethical Committee, Yeungnam University, Republic of Korea. ADMSCs were obtained from C57BL/6 mice (male, 8-week age; Samtako, Republic of Korea). The cells were carefully characterized using specific surface markers and differentiation capacities, according to previous studies [34]. ADMSCs were cultured in MEM-α supplemented with 10% fetal bovine serum (Thermo Fisher Scientific, Waltham, MA, USA) and 1% penicillin/streptomycin 100X (Hyclone Laboratories, South Logan, UT, USA). The cells were used within passages 3–5.

### 3.5. Effect of Isolated Compounds on the Viability of tBHP-Damaged ADMSCs

The stock solution of each compound was prepared in advance by dissolving it in cell culture-grade DMSO (Sigma-Aldrich). To investigate the effect of the compounds, ADMSCs were chemically induced with tBHP (Tokyo Chemical Industry, Tokyo, Japan), and cell viability was measured using a cell counting kit 8 (CCK-8; Dojindo, Molecular Technologies Inc., Rockville, MD, USA). Briefly, ADMSCs were trypsinized and cultured in 96-well plates at a density of 5000 cells/well. The following day, the cells were incubated in a medium containing 100 µM tBHP and the compounds at various concentrations (3–50 µM) for 24 h in a CO_2_ incubator at 37 °C. The cells were then washed twice using phosphate-buffered saline (pH = 7.4; Hyclone Laboratories) and incubated in a medium (120 µl/well) containing 5% CCK-8 reagent for 2 h at 37 °C. The absorbance of the supernatant in a 96-well plate was measured at 450 nm using a plate spectrophotometer (Spark 10M; Tecan, Männedorf, Switzerland). Relative cell viability was calculated by comparing the absorbance of the control and treated cells. The data were analyzed using the GraphPad Prism software version 8.4.2.

## 4. Conclusions

In this study, three new and eleven known sesquiterpenes were isolated from the dried fruits of *Alpinia oxyphylla*. Their structures were elucidated and identified by NMR spectroscopic methods. In the screening for biological activities on adipose-derived mesenchymal stem cells (ADMSCs) of the isolates **1–3**, **5–14,** compounds **8** to **13** effectively prevented oxidative stress-induced ADMSC death. Among these, compounds **11** and **12** showed the highest protective effect. These results supported that *A. oxyphylla* fruit-derived sesquiterpenoids and active compounds enriched extract can be applied to regenerative medicine therapies by prevention of oxidative stress in ADMSCs, which can be differentiated into neurons, osteoblasts, pancreatic cells, etc. Further examination using in vivo degenerative disease models is required in future studies.

## Figures and Tables

**Table 1:** ^1^H-NMR spectroscopic data of compounds **1**–**3.**

	1	2	3
Position	*δ*_H_, mult (*J* in Hz).	*δ*_H_, mult (*J* in Hz)	*δ*_H_, mult (*J* in Hz)
1α	2.30, dd (16.2, 1.3)	2.26, s	5.84, d (1.5)
1β	2.14, dd (16.2, 1.3)		
2			
3	5.90, dt (2.9, 1.3)	5.90, dq (2.9, 1.4)	4.39, dd (5.3, 2.2)
4			2.03, dd (7.1, 5.3)
5	2.37, m	2.83, ddq (13.2, 2.9, 1.4)	
6α	1.54, m	1.55, dd (14.0, 13.2)	1.76, m
6β	2.41, m	2.34, dt (14.0, 2.9)	1.81, t (12.3)
7			2.59, m
8α	1.78, td (14.1, 4.1)	1.71, m	2.07, m
8β	2.19, m	1.91, m	1.32, dd (13.1, 4.5)
9α	1.53, m	1.34, m	2.55, tdd (12.9, 4.5, 1.5)
9β	1.42, td (13.9, 4.1)	1.72, m	2.29, ddd (12.9, 4.5, 2.7)
10			
11			
12a	5.37, t (1.3)	5.08, brs	4.75, t (1.5)
12b	5.21, brs	5.04, t (1.4)	4.72, brs
13	1.84, brs	1.84, m	1.72, brs
14	0.96, brs	0.89, brs	1.28, brs
15	1.93, t (1.3)	1.91, t (1.4)	0.92, d (7.1)
OH			3.26, d (2.2)

The ^1^H-NMR data were acquired using CDCl_3_ at 400 MHz.

**Table 2:** ^13^C-NMR spectroscopic data of compounds **1–3.**

	1	2	3
Position	*δ*_C_, type	*δ*_C_, type	*δ*_C_, type
1	54.1, CH_2_	54.0, CH_2_	119.6, CH
2	198.9, C	199.5, C	199.8, C
3	127.2, CH	127.1, CH	73.4, CH
4	162.0, C	163.7, C	44.0, CH
5	44.5, CH	42.4, CH	41.3, C
6	28.7, CH_2_	28.5, CH_2_	43.7, CH_2_
7	86.7, C	85.1, C	40.1, CH
8	26.7, CH_2_	27.2, CH_2_	34.6, CH_2_
9	37.2, CH_2_	35.2, CH_2_	33.1, CH_2_
10	37.9, C	37.2, C	170.7, C
11	141.5, C	147.8, C	148.3, C
12	118.5, CH_2_	112.5, CH_2_	109.9, CH_2_
13	18.9, CH_3_	18.9, CH_3_	21.1, CH_3_
14	22.1, CH_3_	22.0, CH_3_	9.0, CH_3_
15	17.0, CH_3_	16.2, CH_3_	22.7, CH_3_

The ^13^C-NMR data were acquired using CDCl_3_ at 100 MHz.

## Supplementary Materials

The following are available online.
