# Supplementary material for: Eudesmane and Eremophilane Sesquiterpenes from the Fruits of Alpinia oxyphylla with Protective Effects against Oxidative Stress in Adipose-Derived Mesenchymal Stem Cells
Source: Molecules. 2021 Mar 21;26(6):1762. doi: 10.3390/molecules26061762 (PMC8004014; doi:10.3390/molecules26061762)
Supplement: Supplementary file 1 [file molecules-26-01762-s001.pdf]

Supporting Information

Eudesmane and eremophilane sesquiterpenes from the fruits of *Alpinia oxyphylla* with protective effects against oxidative stress in adipose-derived mesenchymal stem cells

[1] *College of Pharmacy, Yeungnam University, Gyeongsan, Gyeongsangbukdo 38541, Republic of Korea*

[2] *College of Pharmacy, Graduate School of Pharmaceutical Sciences, Ewha Womans University, Seoul 03760, Republic of Korea*

[3] *Advanced Radiation Technology Institute, Korea Atomic Energy Research Institute, Jeollabuk-do, Jeongeup-si 56212, Republic of Korea*

*Authors for correspondence

E-mail addresses: jwnam@yu.ac.kr (J.-W. Nam), yuny@ewha.ac.kr (E. K. Seo)

Table of contents

Figure	S1.	^{1}H NMR (400 MHz, $CDCl_3$) of compound **1**……………………………………………...	3
Figure	S2.	^{13}C NMR (100 MHz, $CDCl_3$) of compound **1**………………………………………..	4
Figure	S3.	DEPT-135 (100 MHz, $CDCl_3$) of compound **1**…………………...................................	5
Figure	S4.	^{1}H-^{1}H COSY NMR ($CDCl_3$) of compound **1**……………………………………………	6
Figure	S5.	^{1}H-^{13}C HSQC NMR ($CDCl_3$) of compound **1**……………………………………......	7
Figure	S6.	^{1}H-^{13}C HMBC NMR ($CDCl_3$) of compound **1**…………………………………….....	8
Figure	S7.	^{1}H-^{1}H NOESY NMR ($CDCl_3$) of compound **1**……………………….........................	9
Figure	S8.	HR-ESIMS of compound **1** ………………………………………………………………..	10
Figure	S9.	^{1}H NMR (400 MHz, $CDCl_3$) of compound **2**……………………………………………...	11
Figure	S10.	^{13}C NMR (100 MHz, $CDCl_3$) of compound **2**………………………………………..	12
Figure	S11.	DEPT-135 (100 MHz, $CDCl_3$) of compound **2**……………………............................	13
Figure	S12.	^{1}H-^{1}H COSY NMR ($CDCl_3$) of compound **2**……………………………………………	14
Figure	S13.	^{1}H-^{13}C HSQC NMR ($CDCl_3$) of compound **2**……………………………………......	15
Figure	S14.	^{1}H-^{13}C HMBC NMR ($CDCl_3$) of compound **2**…………………………………….....	16
Figure	S15.	^{1}H-^{1}H NOESY NMR ($CDCl_3$) of compound **2**……………………….........................	17
Figure	S16.	HR-ESIMS of compound **2** ………………………………………………………………..	18
Figure	S17.	^{1}H NMR (400 MHz, $CDCl_3$) of compound **3**……………………………………………...	19
Figure	S18.	^{13}C NMR (100 MHz, $CDCl_3$) of compound **3**………………………………………..	20
Figure	S19.	DEPT-135 (100 MHz, $CDCl_3$) of compound **3**…………………...................................	21
Figure	S20.	^{1}H-^{1}H COSY NMR ($CDCl_3$) of compound **3**……………………………………………	22
Figure	S21.	^{1}H-^{13}C HSQC NMR ($CDCl_3$) of compound **3**……………………………………......	23
Figure	S22.	^{1}H-^{13}C HMBC NMR ($CDCl_3$) of compound **3**…………………………………….....	24
Figure	S23.	^{1}H-^{1}H NOESY NMR ($CDCl_3$) of compound **3**……………………….........................	25
Figure	S24.	HR-ESIMS of compound **3** ………………………………………………………………..	26

Figure S1. ^{1}H NMR (400 MHz, $CDCl_3$) of compound **1**

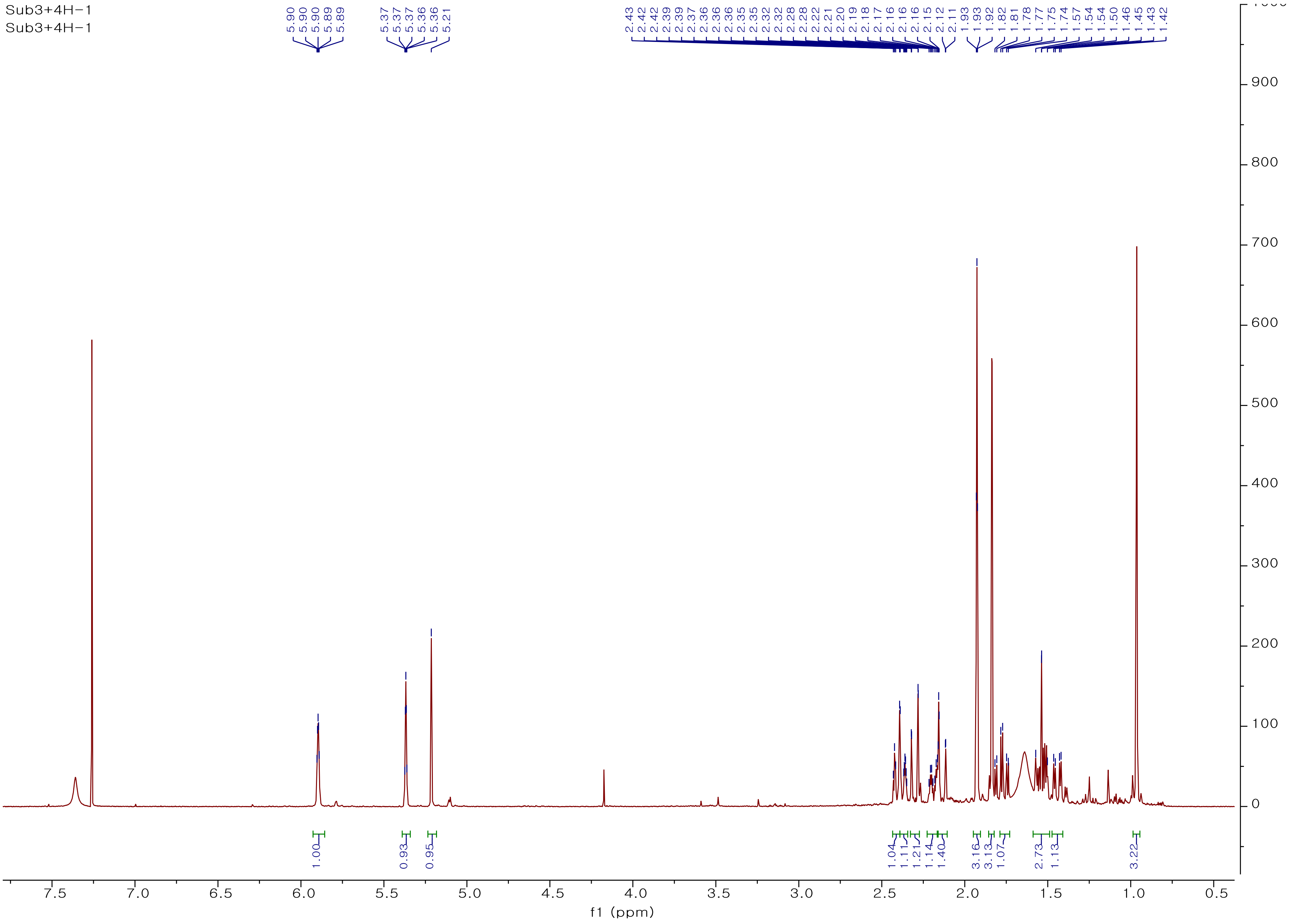

Figure S2. ^{13}C NMR (100 MHz, $CDCl_3$) of compound **1**

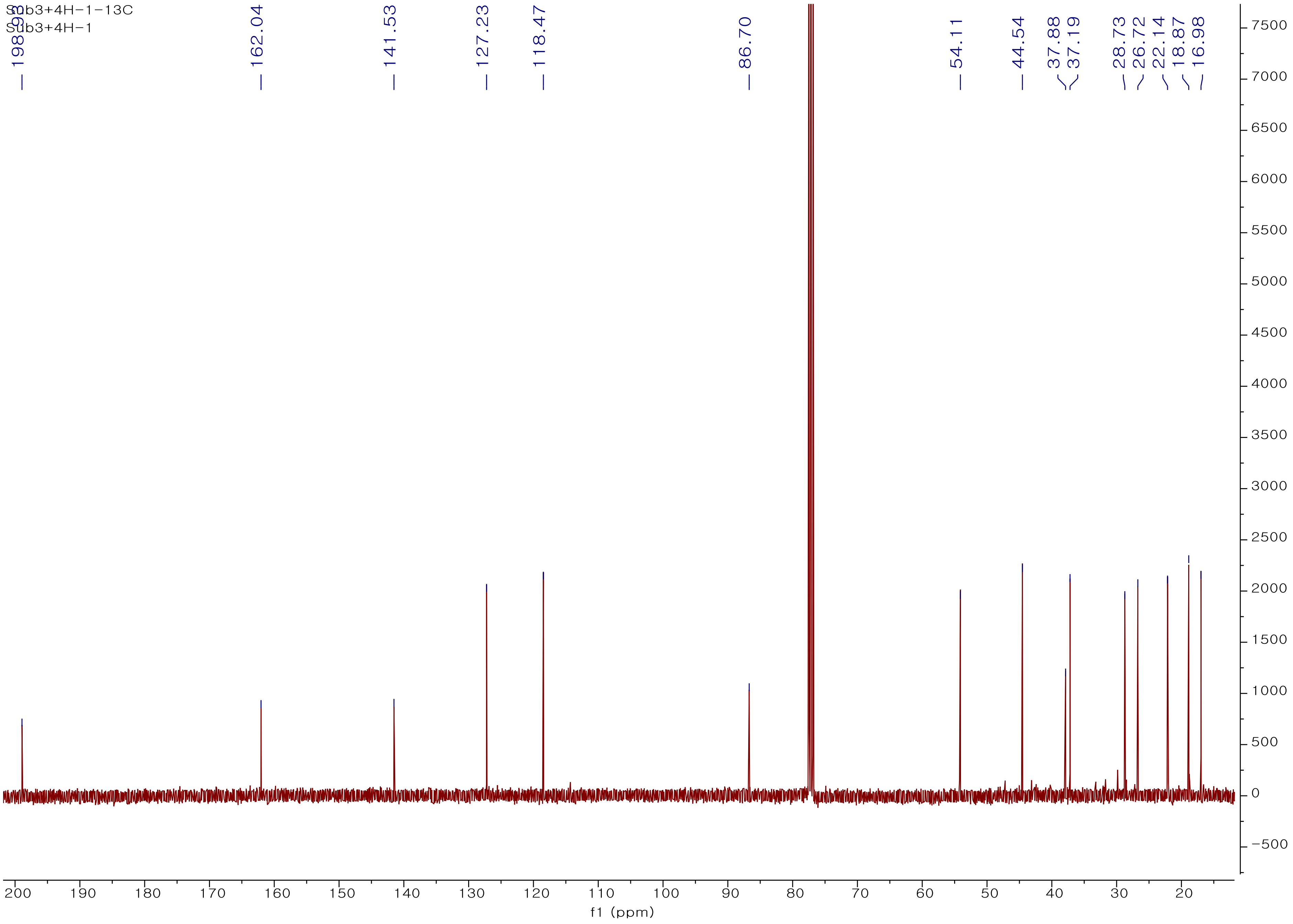

Figure S3. DEPT-135 NMR (100 MHz, $CDCl_3$) of compound **1**

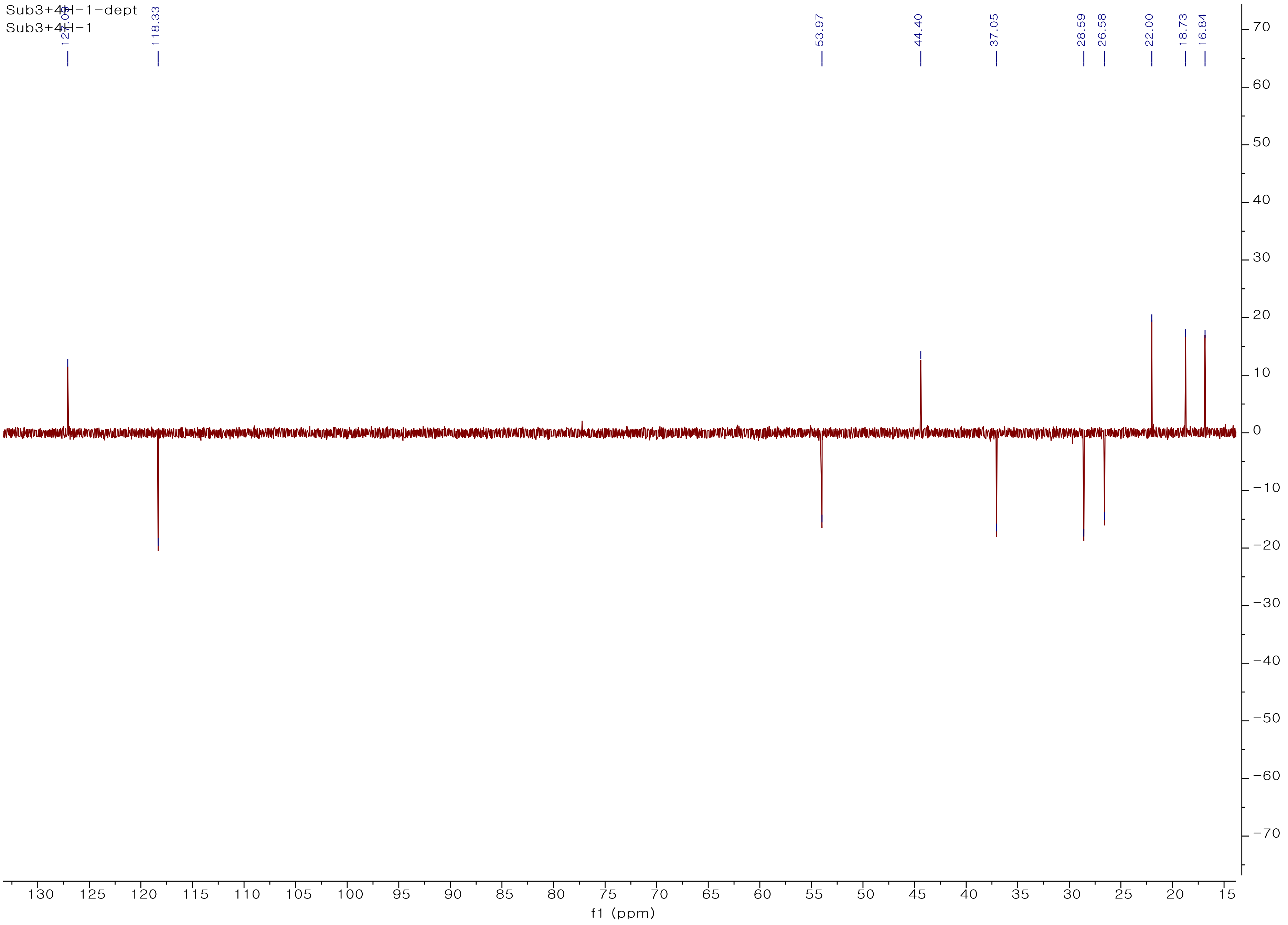

Figure S4. ^{1}H-^{1}H COSY NMR ($CDCl_3$) of compound **1**

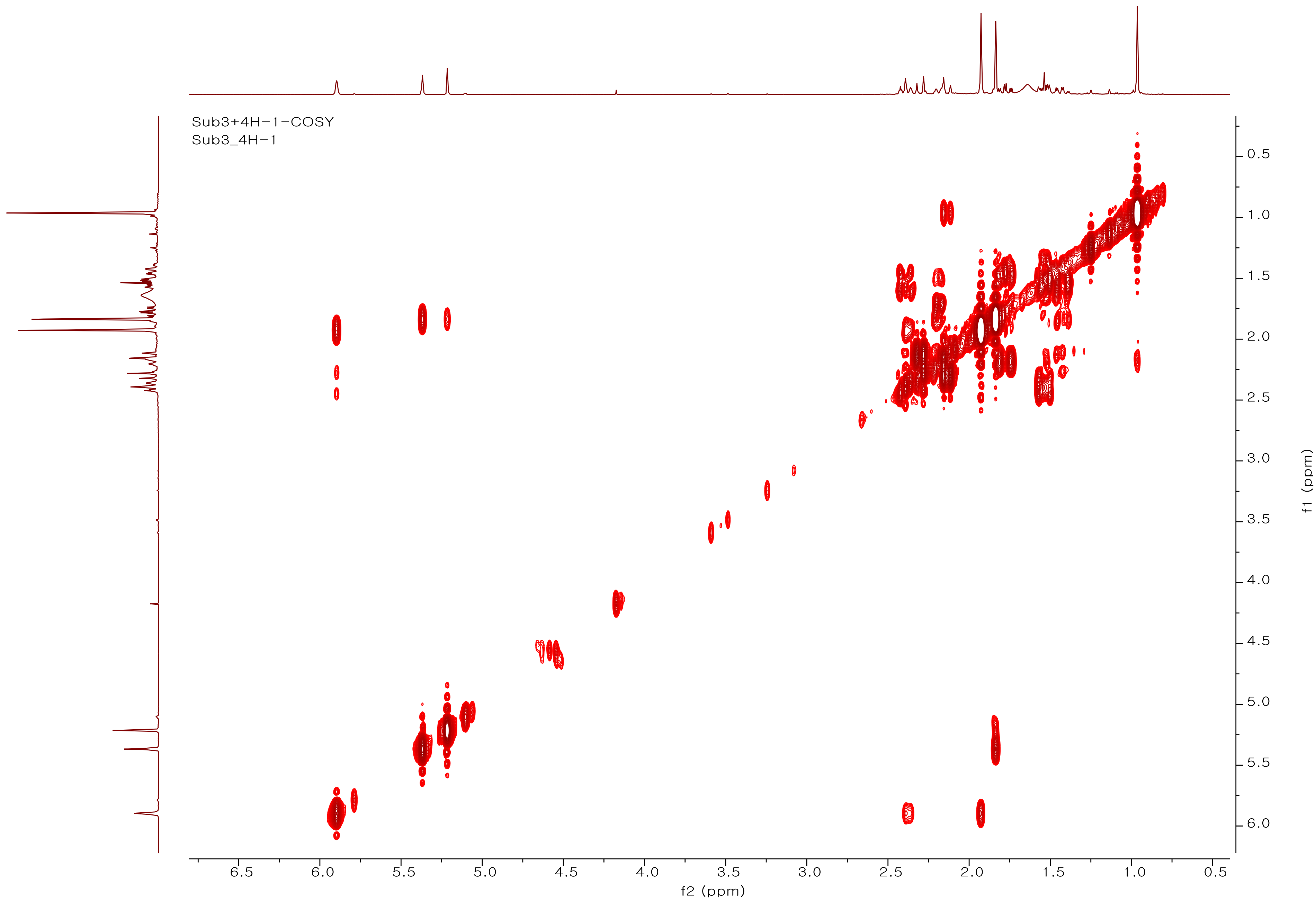

Figure S5. ^{1}H-^{13}C HSQC NMR ($CDCl_3$) of compound **1**

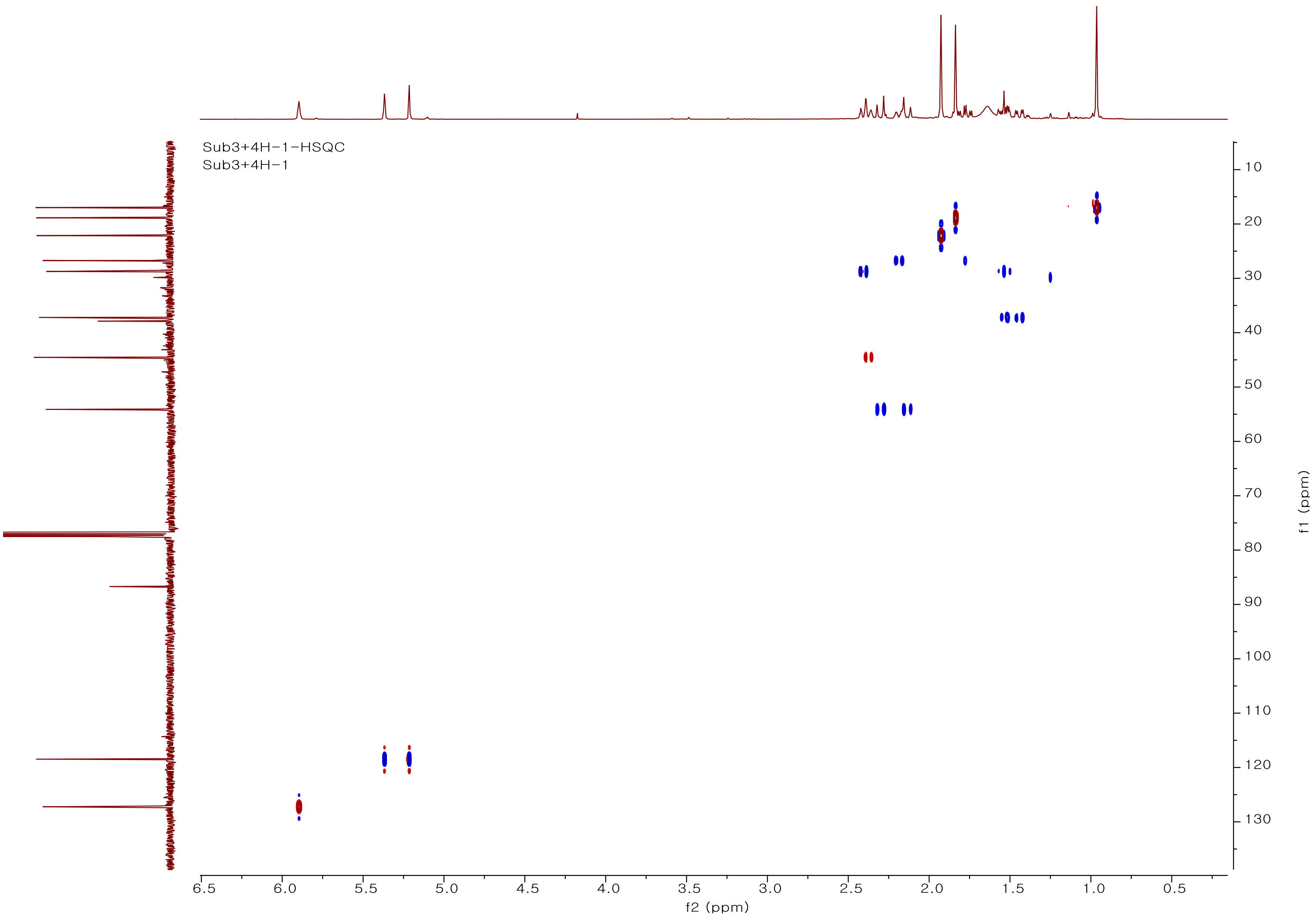

Figure S6. ^{1}H-^{13}C HMBC NMR ($CDCl_3$) of compound **1**

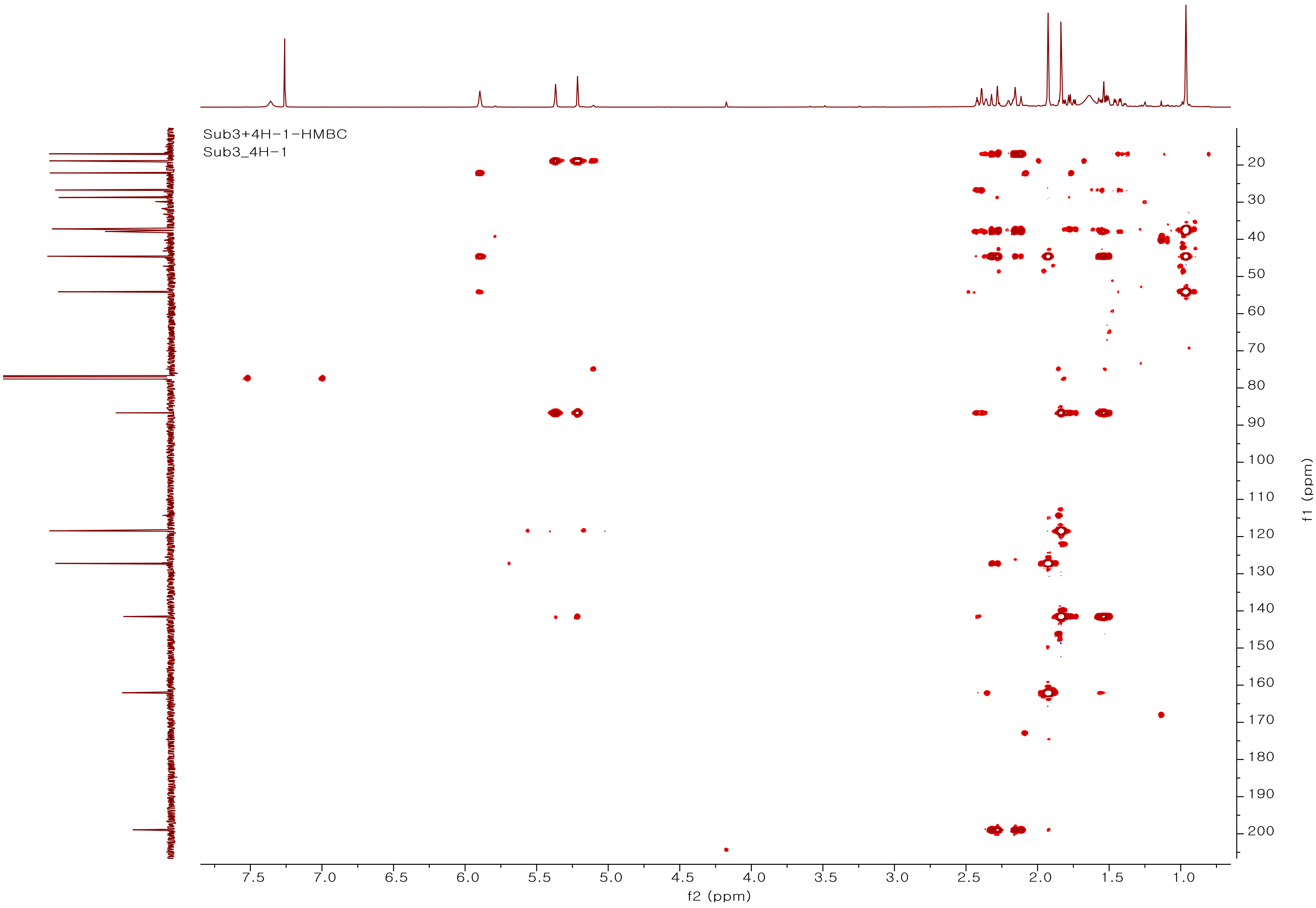

Figure S7. ^{1}H-^{1}H NOESY NMR ($CDCl_3$) of compound **1**

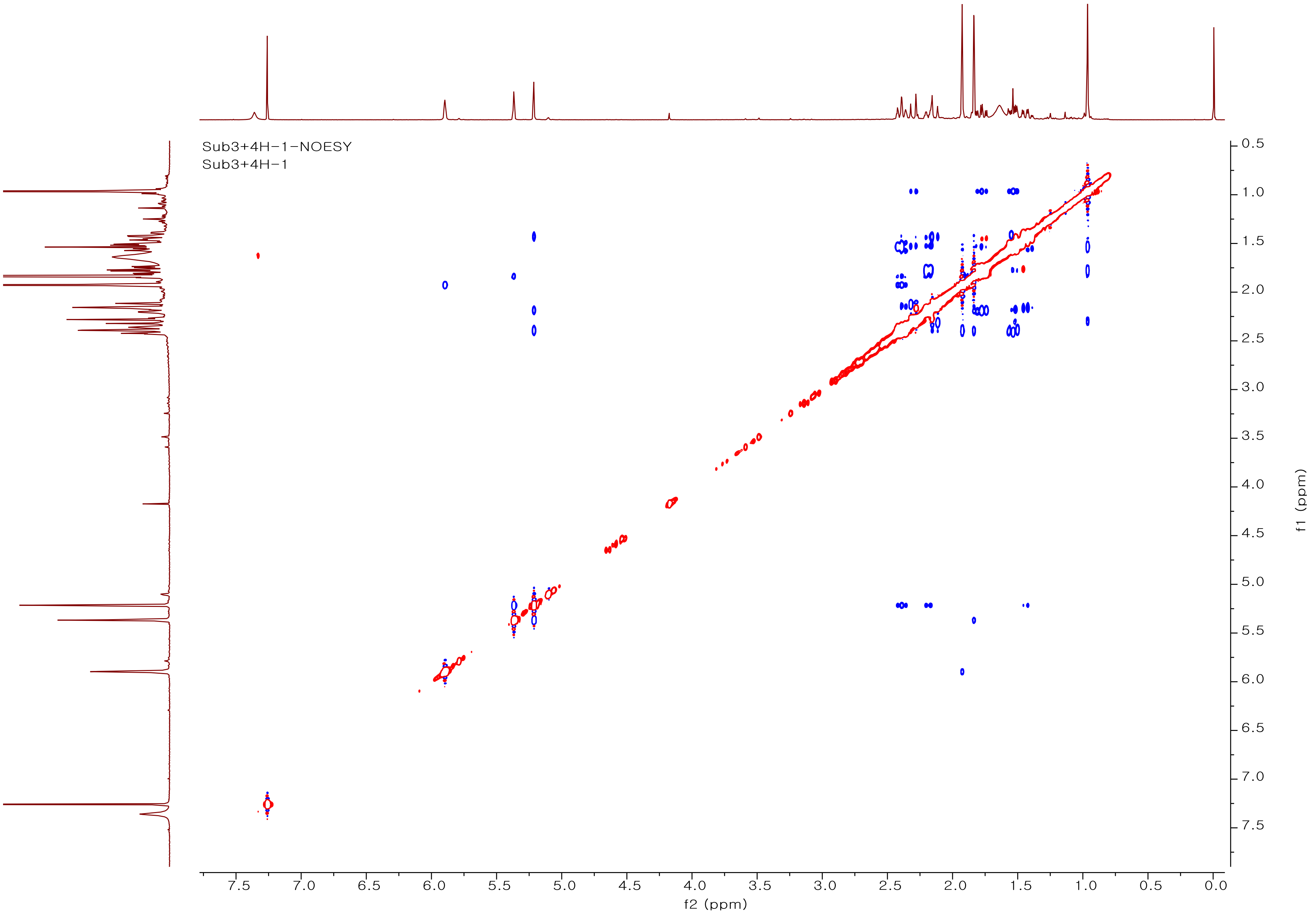

Figure S8. HR-ESIMS of compound **1**

Data File	EA323H-52-9-k1(p)01.d	Sample Name	Sample1
Sample Type	Sample	Position	P1-A3
Instrument Name	Instrument 1	User Name	
Acq Method	B213-nocolumn.m	Acquired Time	1/15/2020 3:58:58 PM
IRM Calibration Status	Success	DA Method	kkksll.m
Comment	1, 110V		

Sample Group		Info.	
Stream Name	LC 1	Acquisition SW Version	6200 series TOF/6500 series Q-TOF B.08.00 (B8058.0)

Compound Table

Compound Label	RT	Mass	MFG Formula	MFG Diff (ppm)
Cpd 365: C15 H22 O3	0.214	250.157	C15 H22 O3	-0.51

Compound Label	*m/z*	RT	Algorithm	Mass
Cpd 365: C15 H22 O3	251.1643	0.214	Find by Molecular Feature	250.157

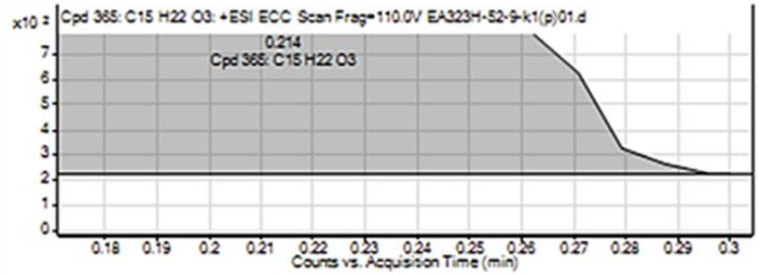

MFE MS Spectrum

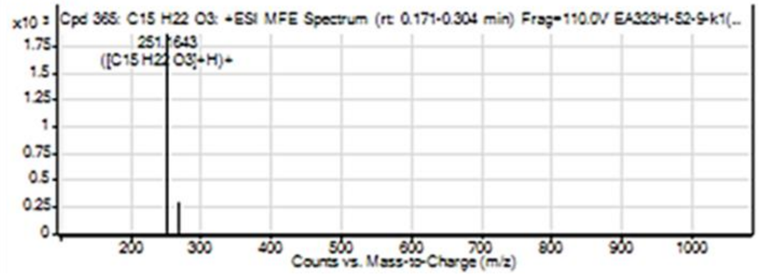

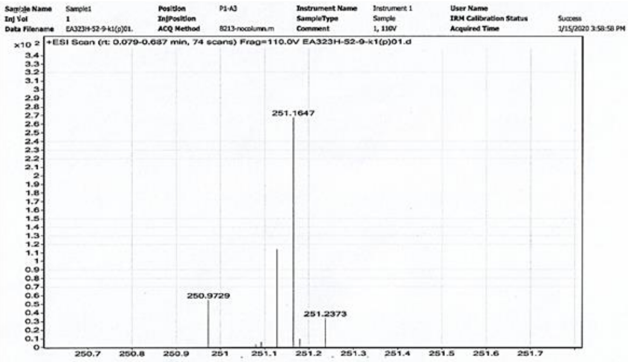

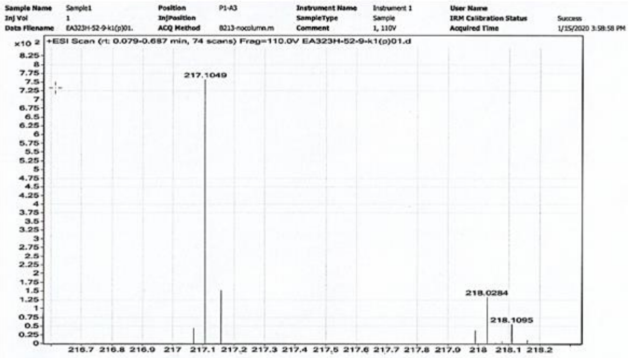

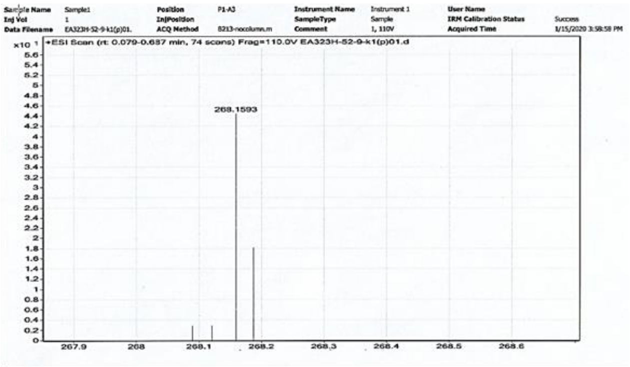

Figure S9. ^1H NMR (400 MHz, $CDCl_3$) of compound **2**

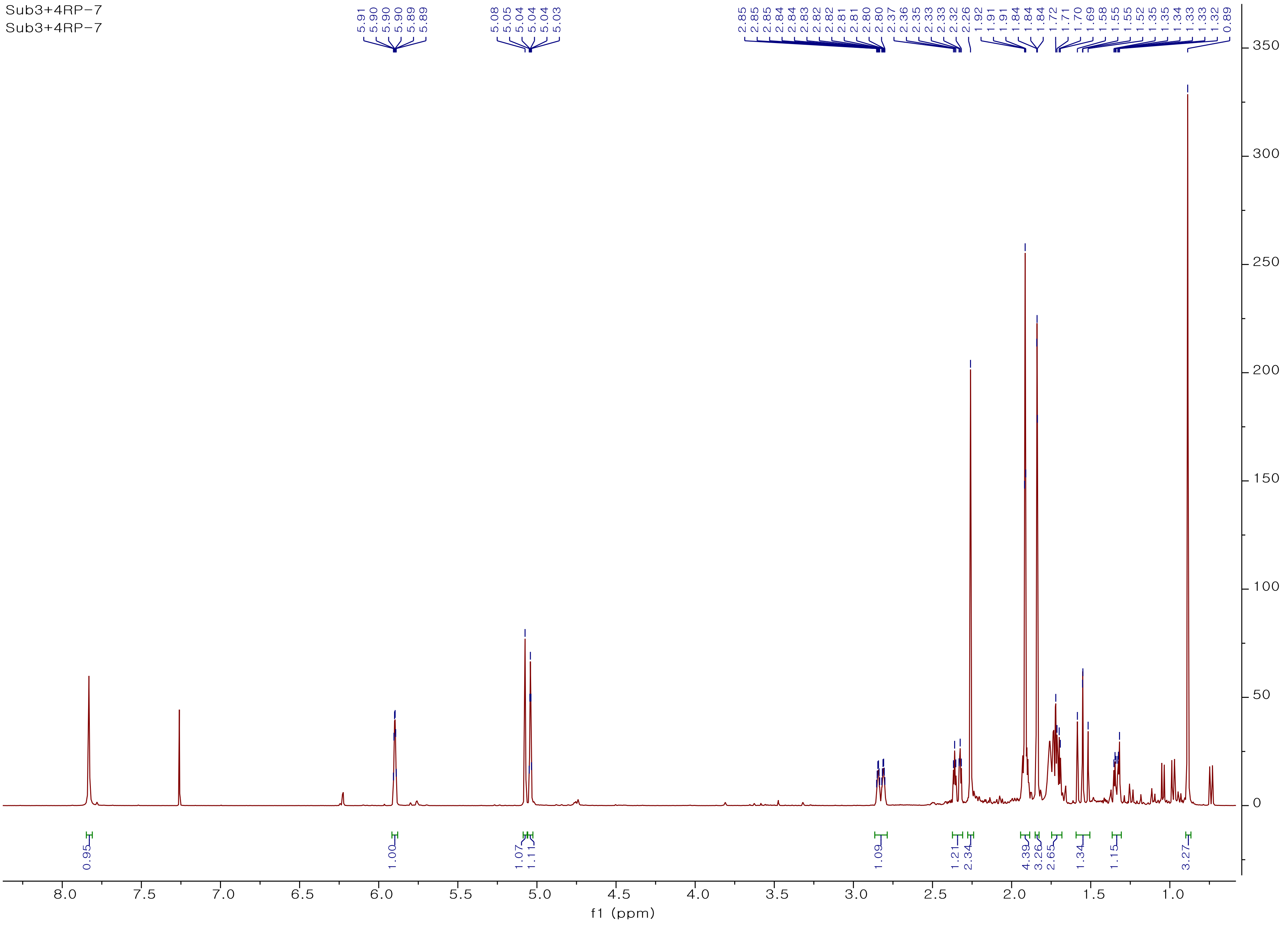

Figure S10. ^{13}C NMR (150 MHz, $CDCl_3$) of compound **2**

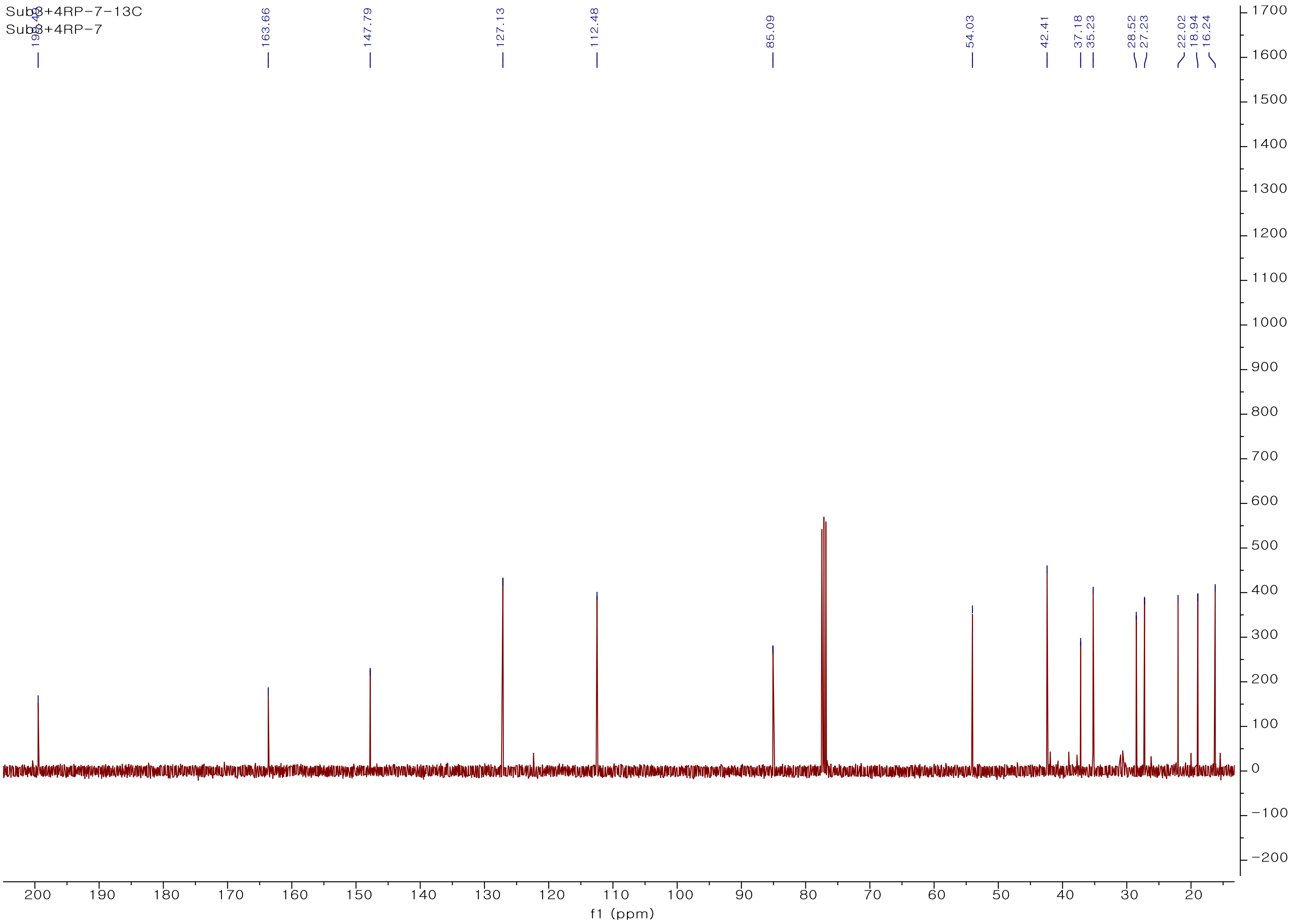

Figure S11. DEPT-135 NMR (150 MHz, $CDCl_3$) of compound **2**

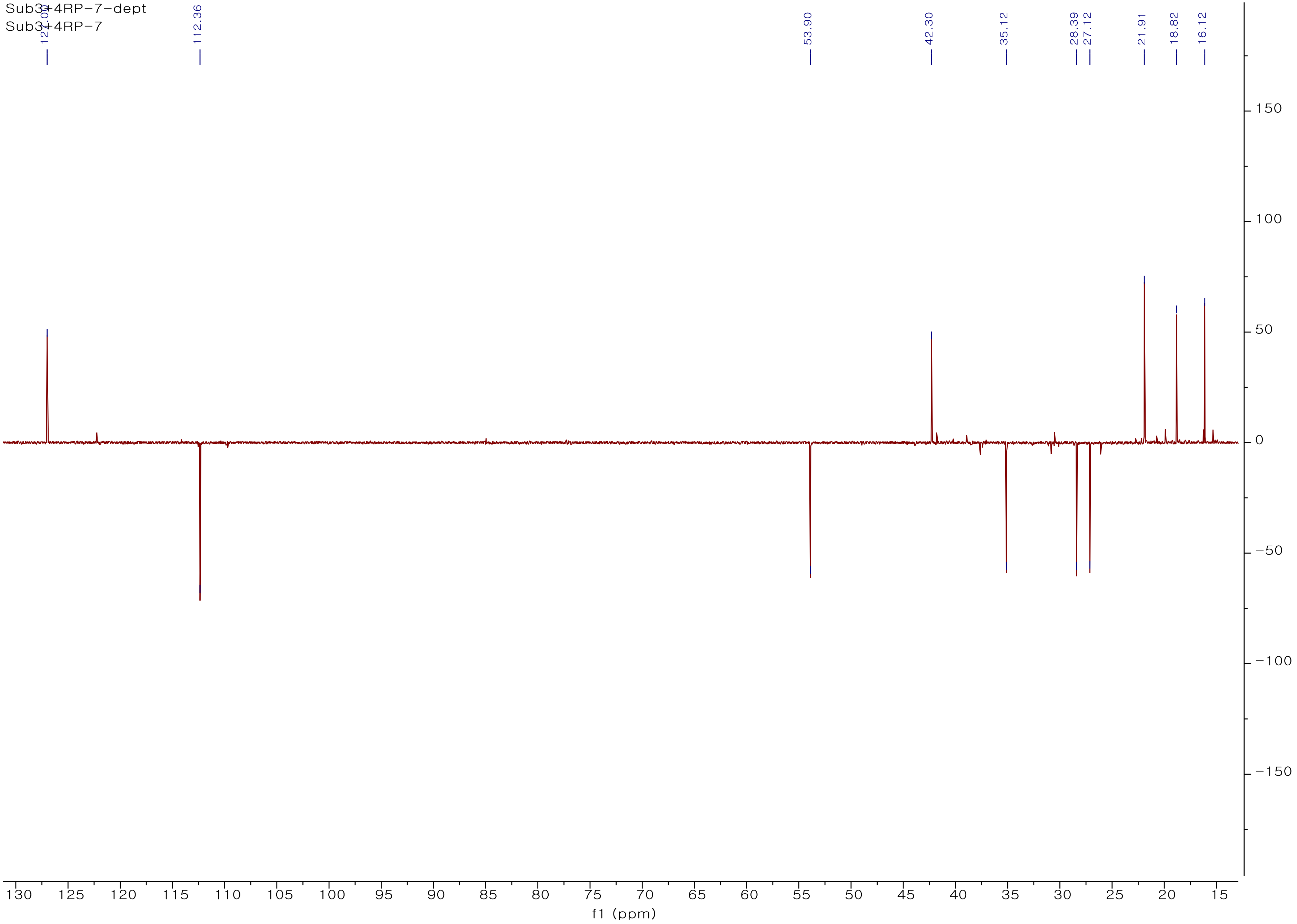

Figure S12. ${}^{1}H$-${}^{1}H$ COSY NMR ($CDCl_3$) of compound **2**

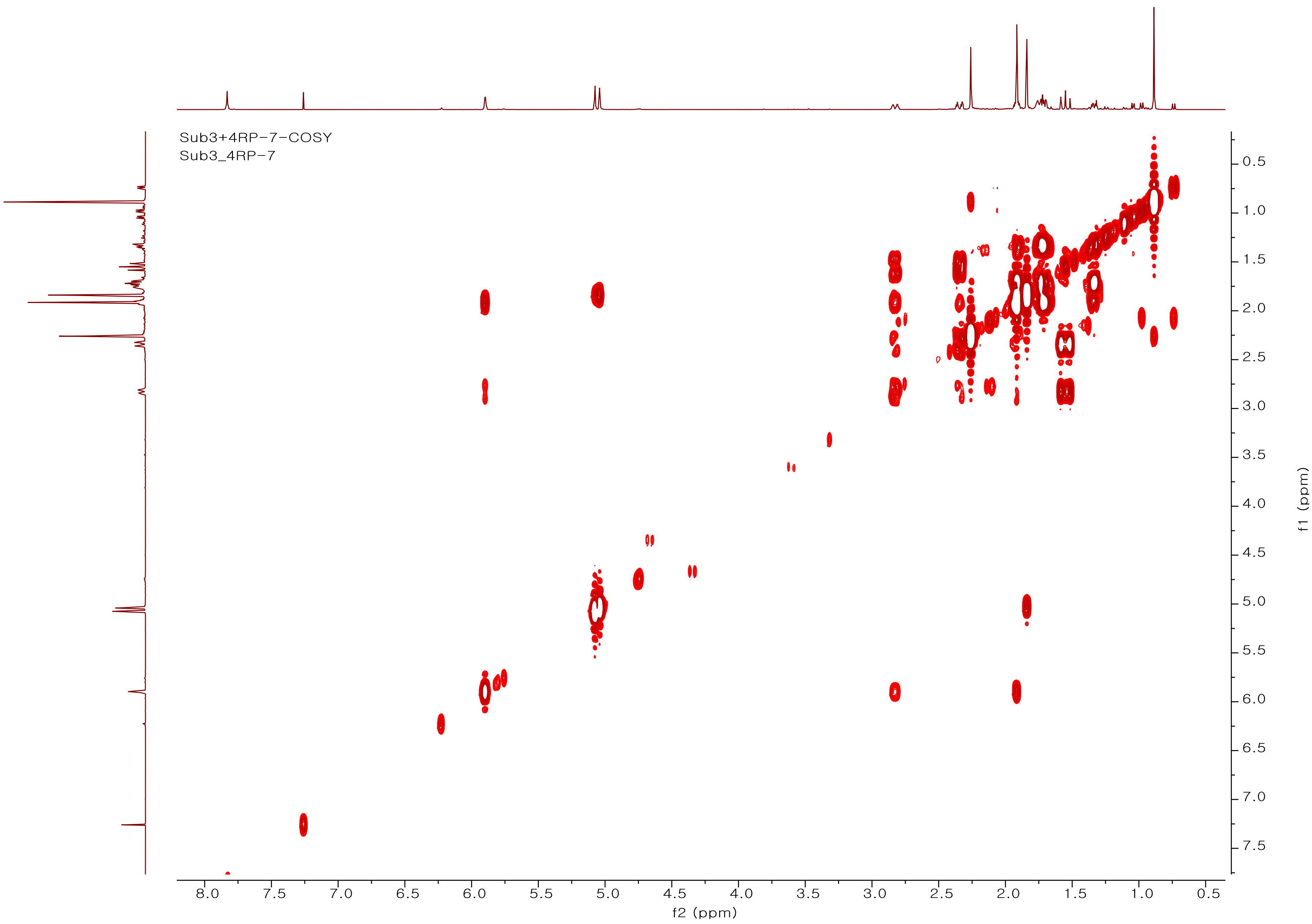

Figure S13. ^{1}H-^{13}C HSQC NMR ($CDCl_3$) of compound **2**

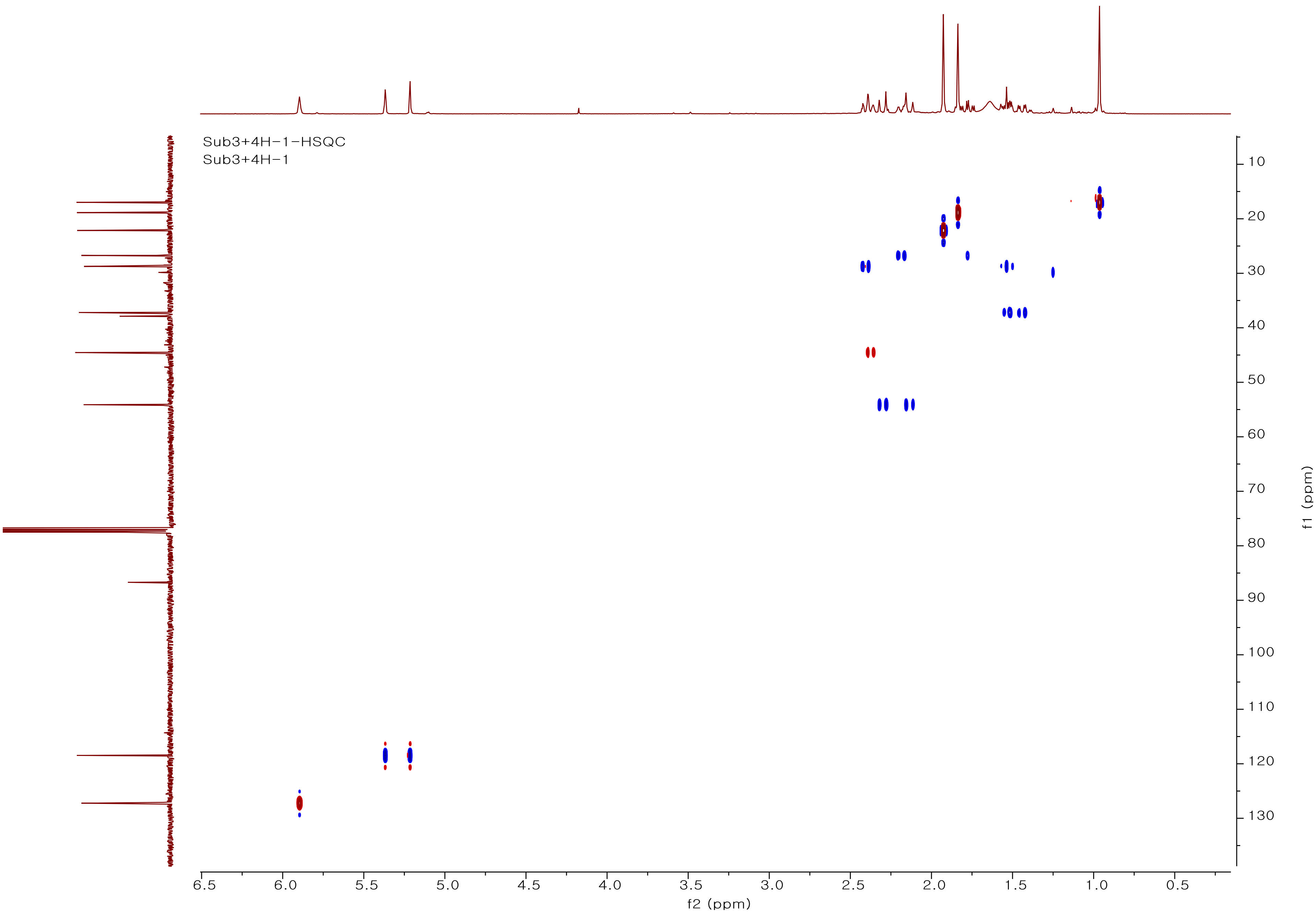

Figure S14. ^{1}H-^{13}C HMBC NMR ($CDCl_3$) of compound **2**

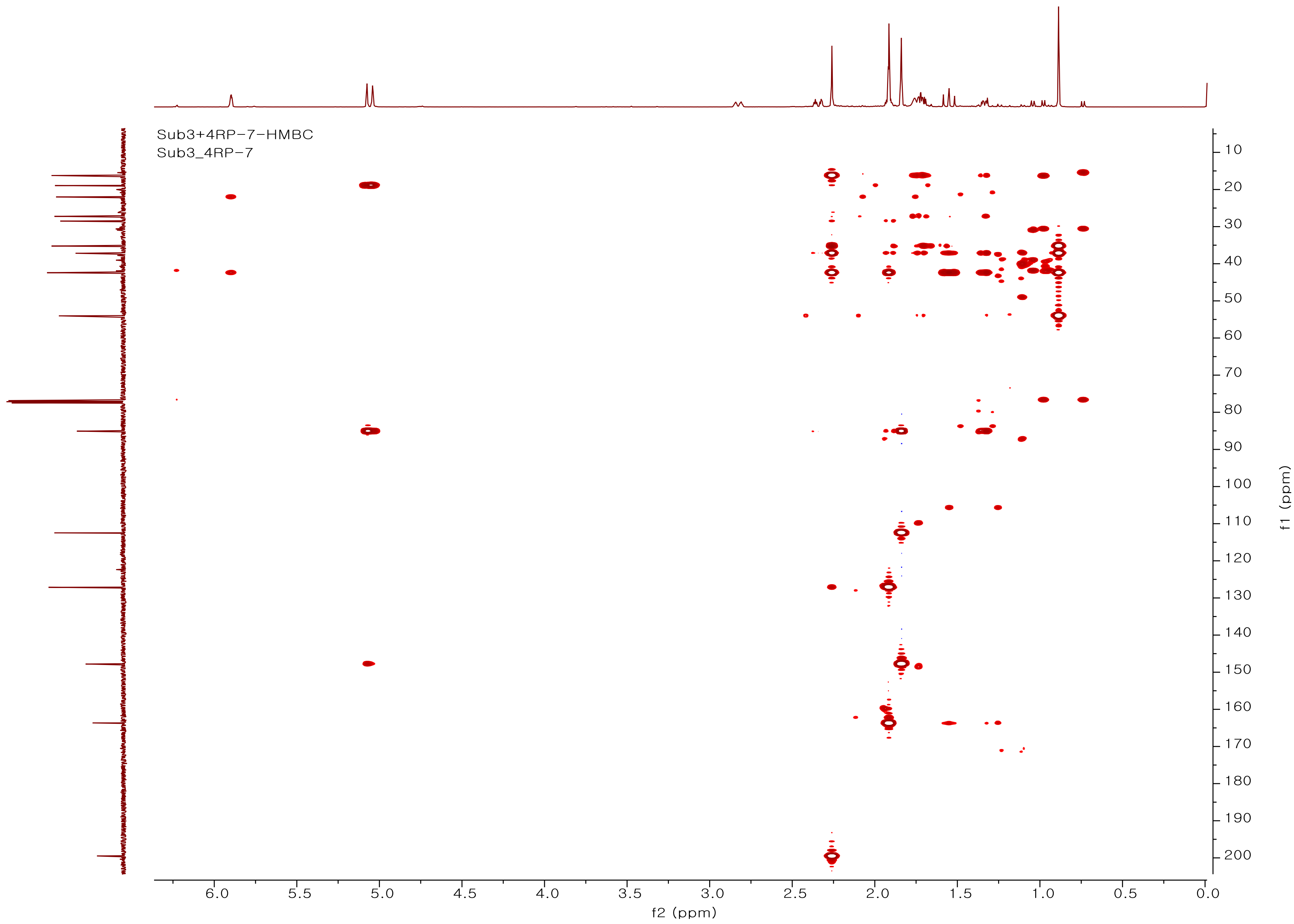

Figure S15. ^{1}H-^{1}H NOESY NMR ($CDCl_3$) of compound **2**

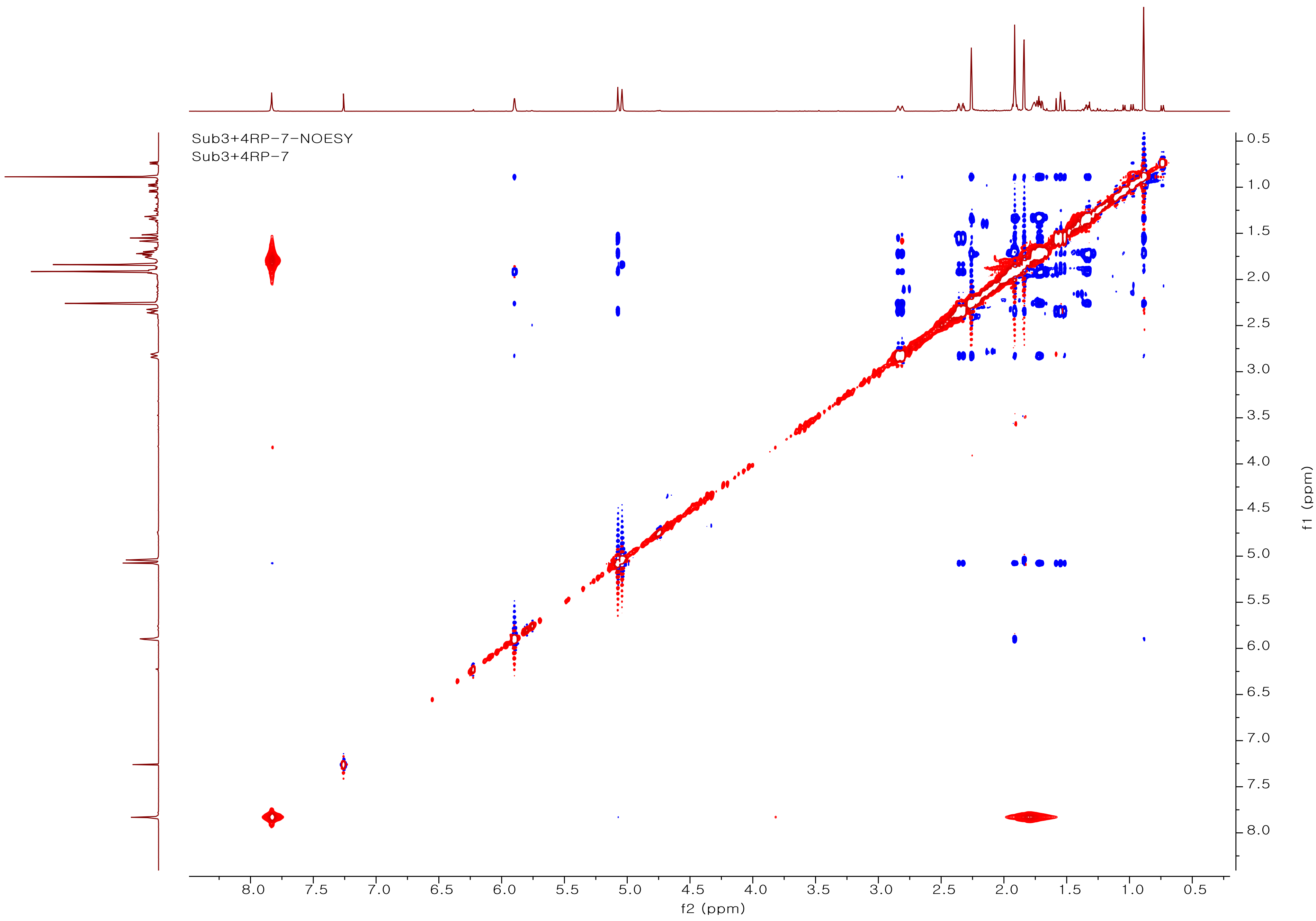

Figure S16. HR-ESIMS of compound **2**

Qualitative Analysis Report

**Data Filename**	EA323H-52-8-k7(20)(p)03.d	**Sample Name**	Sample1
**Sample Type**	Sample	**Position**	P1-F6
**Instrument Name**	Instrument 1	**User Name**	
**Acq Method**	B213-nocolumn.m	**Acquired Time**	1/12/2021 3:32:05 PM
**IRM Calibration Status**	Success	**DA Method**	kikisil.m
**Comment**	2, 110V		

Sample Group **Info.**

Stream Name LC 1 **Acquisition SW** 6200 series TOF/6500 series **Version** Q-TOF B.08.00 (B8058.0)

User Chromatograms

Fragmentor Voltage 110 **Collision Energy** 0 **Ionization Mode** ESI

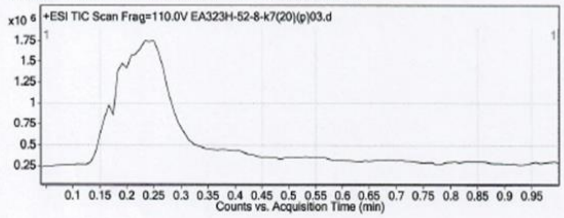

User Spectra

Fragmentor Voltage	Collision Energy	Ionization Mode
110	0	ESI

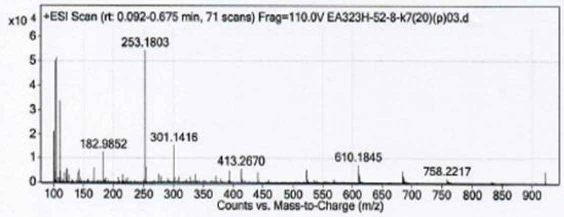

Peak List

m/z	z	Abund
100.076	1	21264.58
102.0344	1	50220.52
105.0426	1	51476.7
110.0204	1	33795.04
167.0129	1	5953.82
182.9852	1	12676.95
253.1803	1	54450.91
254.1837	1	6363.92
301.1416	1	15500.86
610.1845	1	7350.04

Compounds

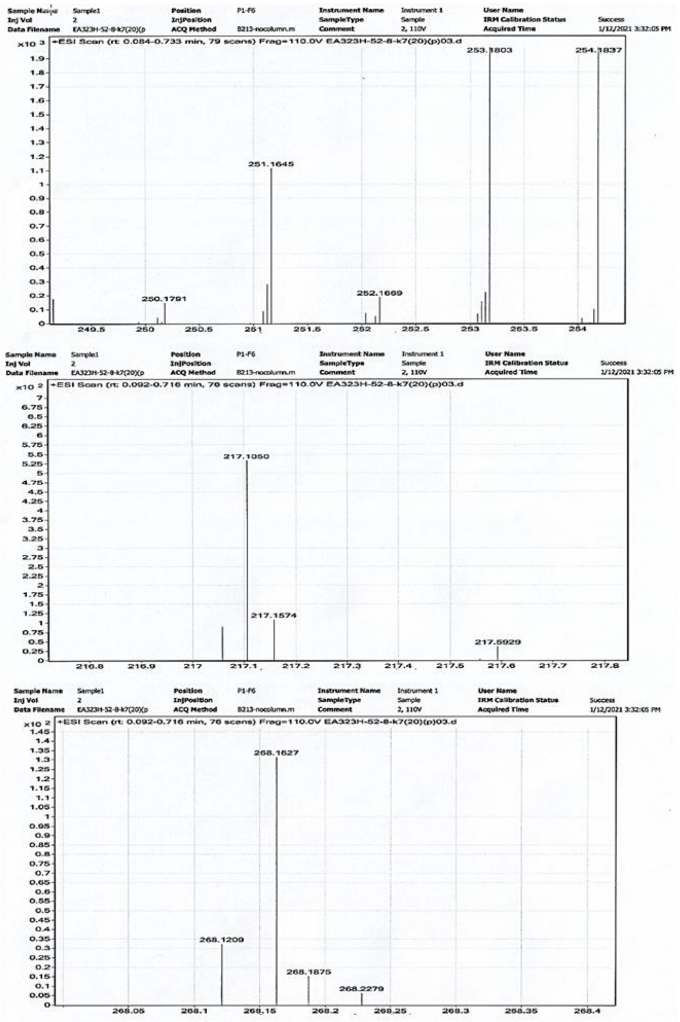

Figure S17. ^{1}H NMR (400 MHz, $CDCl_3$) of compound **3**

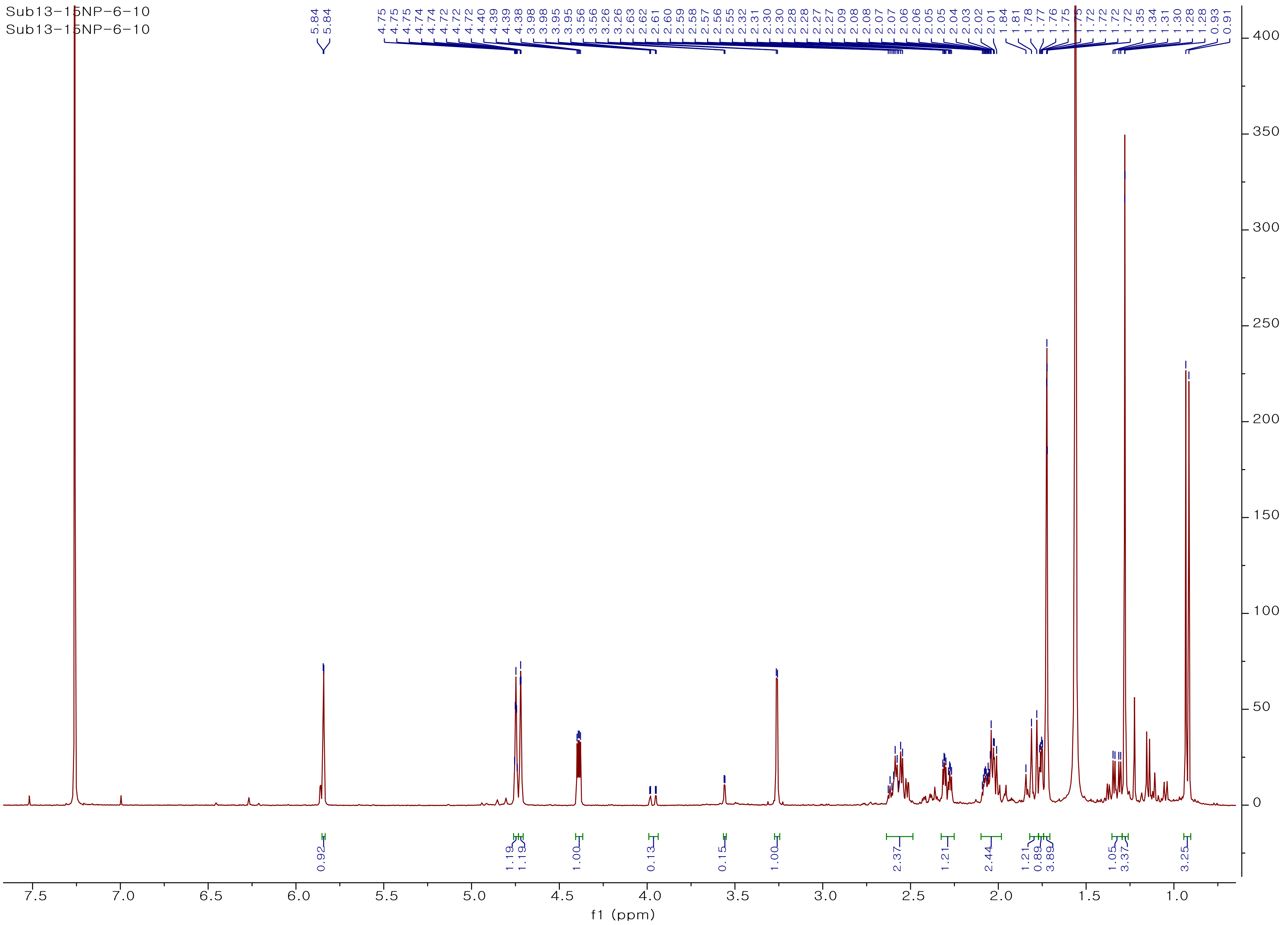

Figure S18. ^{13}C NMR (150 MHz, $CDCl_3$) of compound **3**

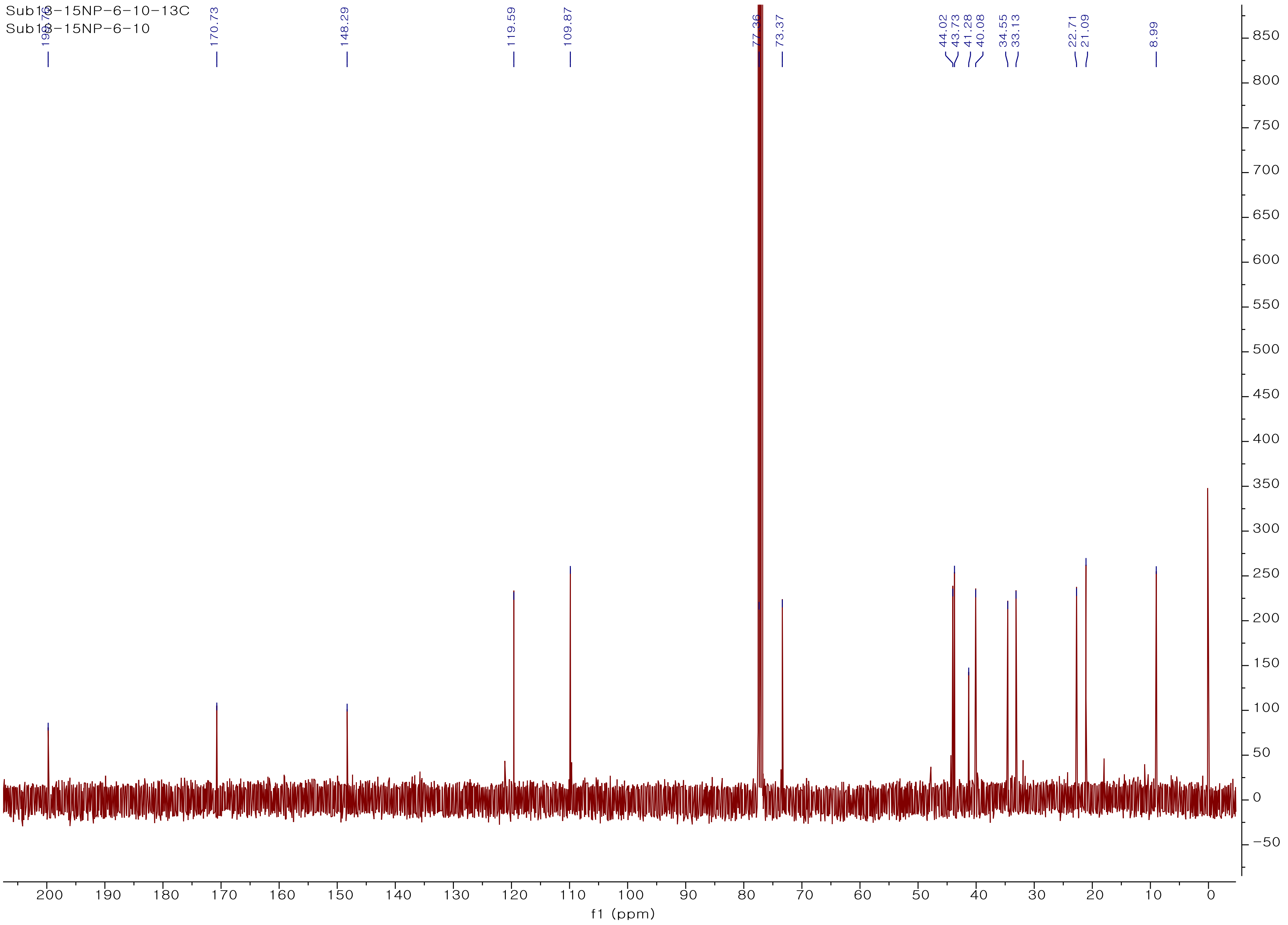

Figure S19. DEPT-135 NMR (150 MHz, CDCl$_3$) of compound **3**

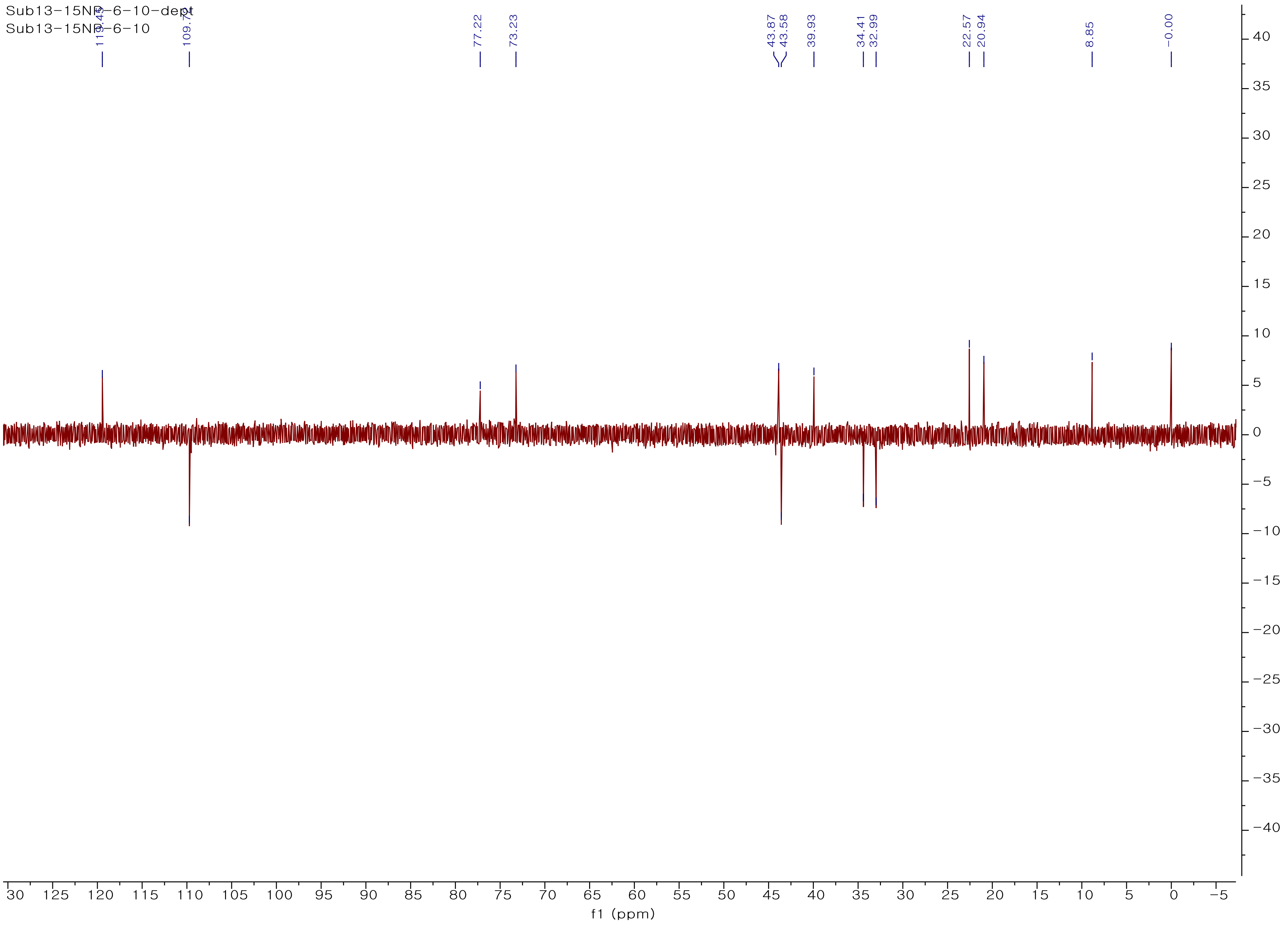

Figure S20. ^{1}H-^{1}H COSY NMR ($CDCl_3$) of compound **3**

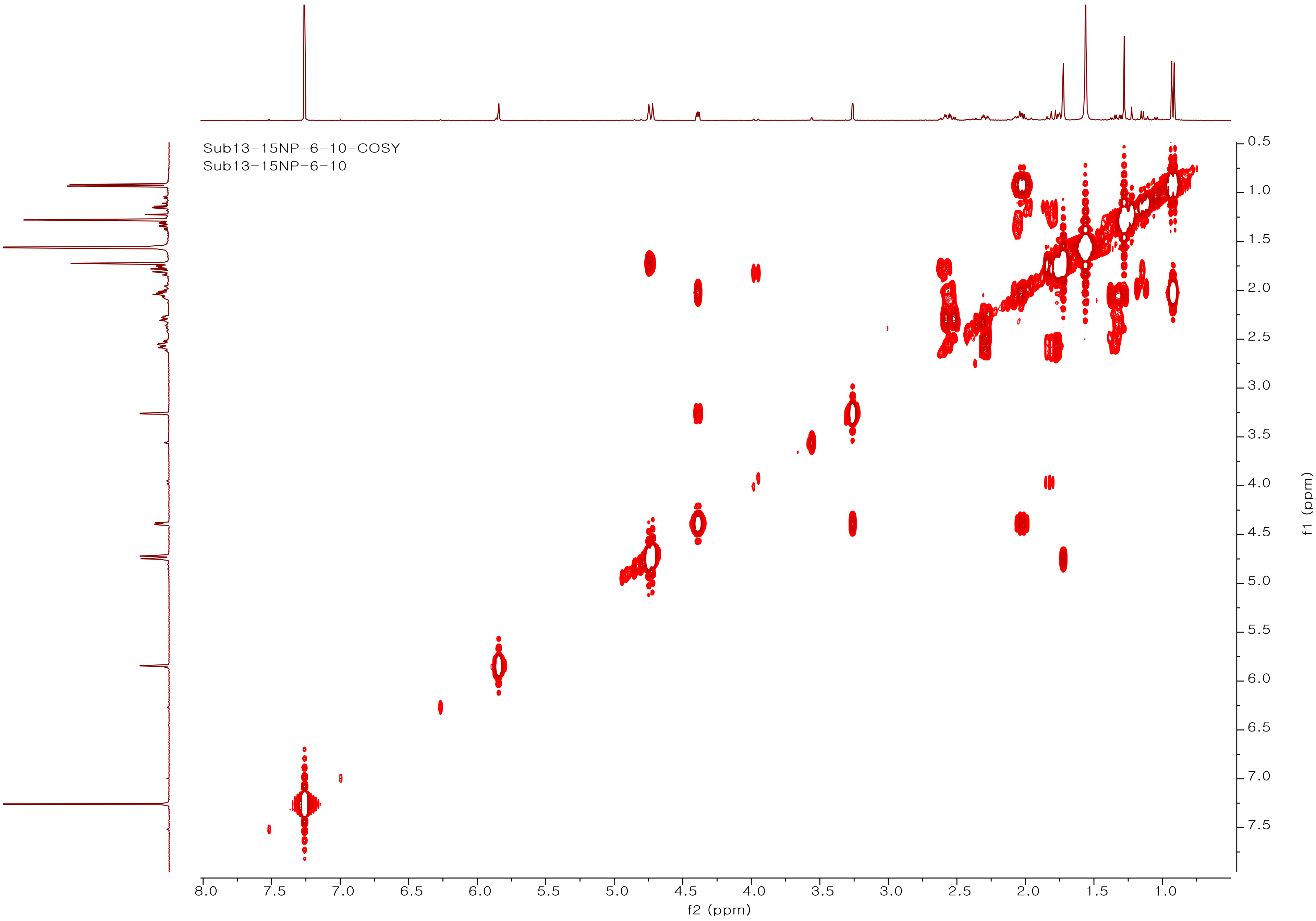

Figure S21. ^{1}H-^{13}C HSQC NMR ($CDCl_3$) of compound **3**

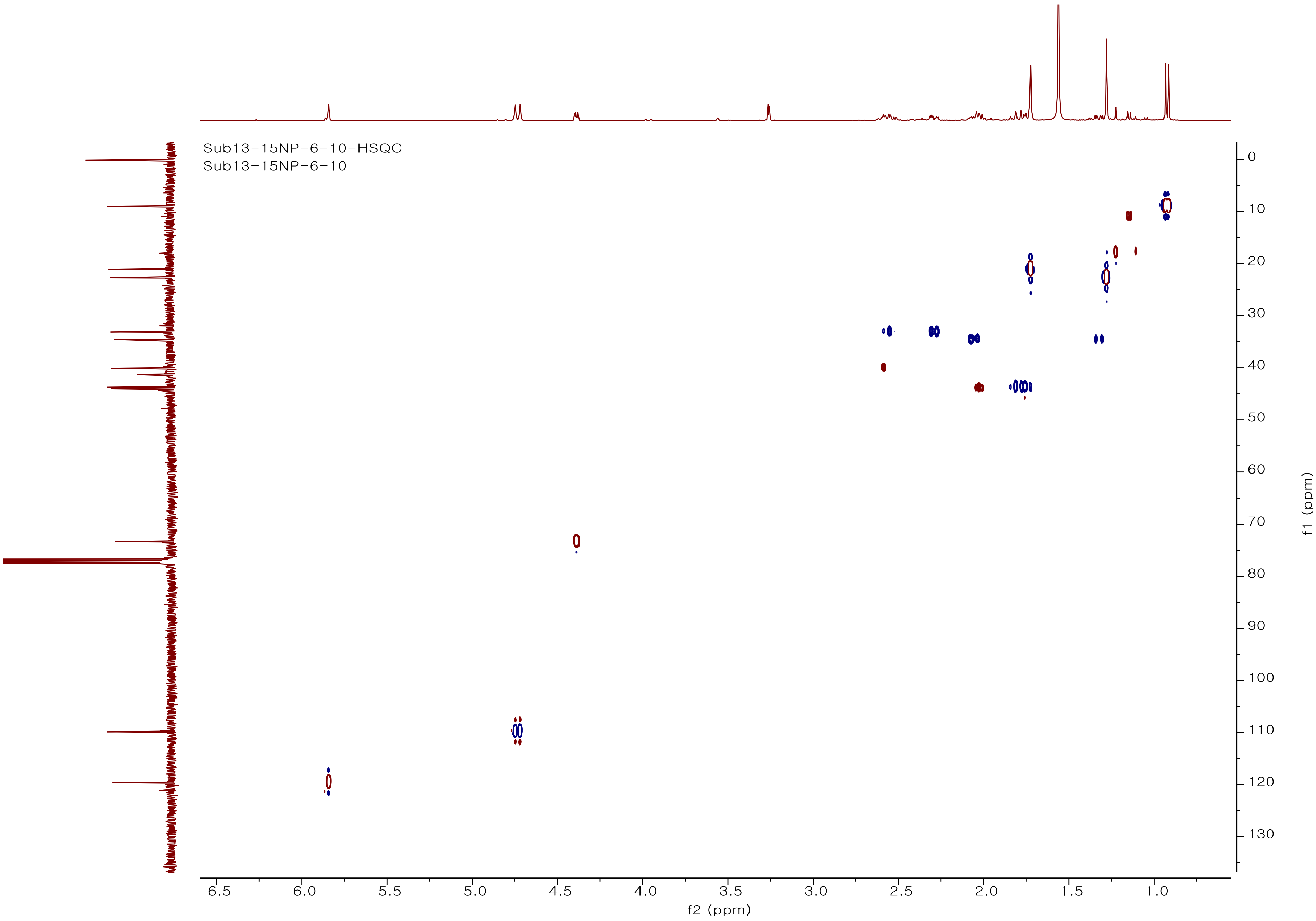

Figure S22. ^{1}H-^{13}C HMBC NMR ($CDCl_3$) of compound **3**

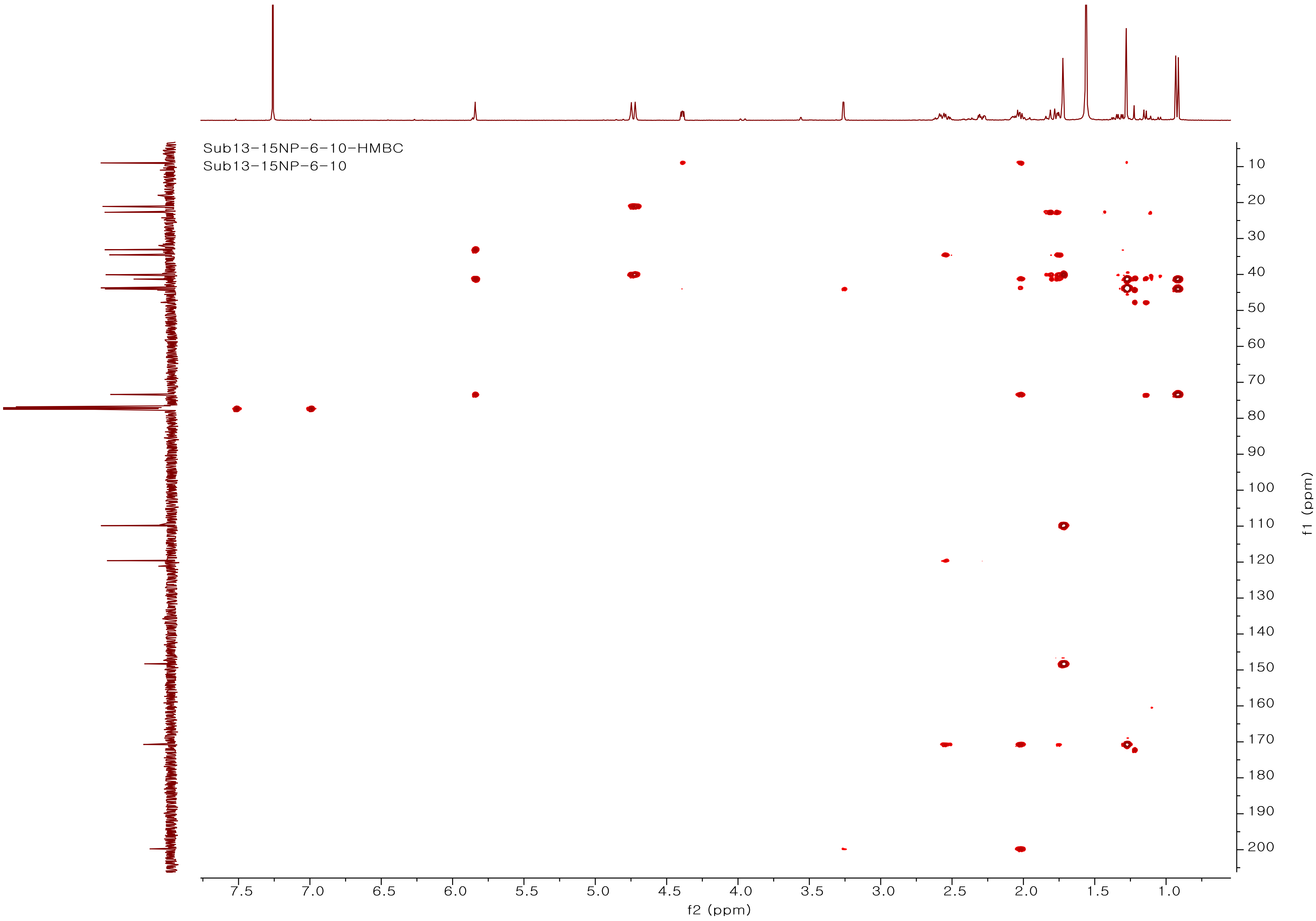

Figure S23. ^1H-^1H NOESY NMR ($CDCl_3$) of compound **3**

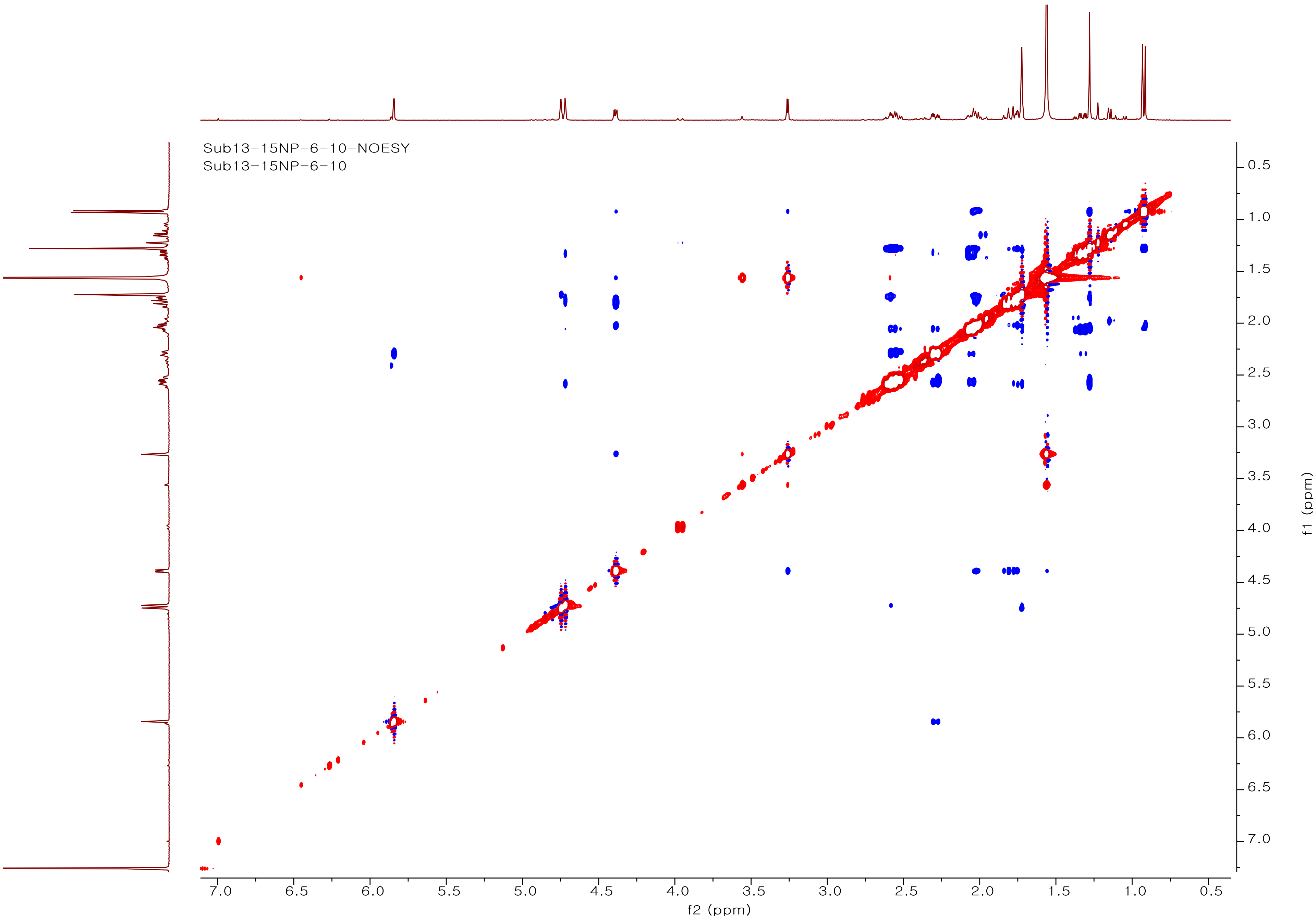

Figure S24. HR-ESIMS of compound **3**

**Data File**	EA323H-52-11-k10(p)01.d	**Sample Name**	Sample1
**Sample Type**	Sample	**Position**	P1-A2
**Instrument Name**	Instrument 1	**User Name**	
**Acq Method**	B213-nocolumn.m	**Acquired Time**	1/15/2020 3:45:45 PM
**IRM Calibration Status**	Success	**DA Method**	kikisil.m
**Comment**	0.1, 110V		

**Sample Group**		**Info.**	
**Stream Name**	LC 1	**Acquisition SW Version**	6200 series TOF/6500 series Q-TOF B.08.00

Compound Table

Compound Label	RT	Mass	Abund	Formula	Tgt Mass	Diff (ppm)
Cpd 1: C15 H22 O2	0.193	234.1617	3592	C15 H22 O2	234.162	-1.15

Compound Label	*m/z*	RT	Algorithm	Mass
Cpd 1: C15 H22 O2	235.1692	0.193	Find By Formula	234.1617

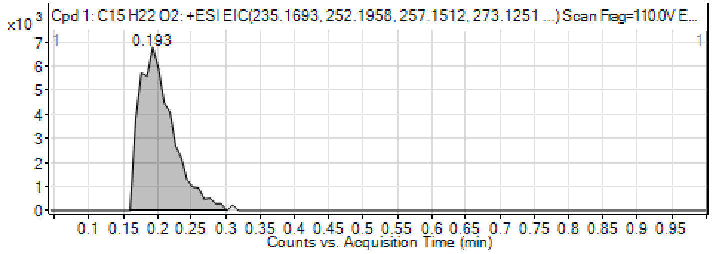

MS Spectrum

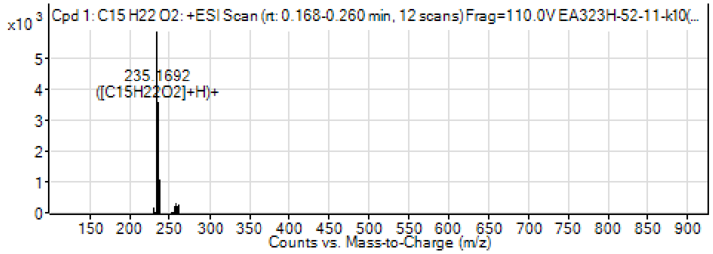

MS Zoomed Spectrum

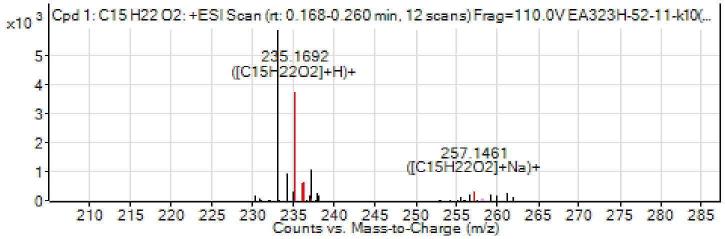

MS Spectrum Peak List

m/z	*Calc m/z*	Diff(ppm)	z	Abund	Formula	Ion
235.1692	235.1693	0.34	1	3591.88	C15H22O2	(M+H)+
236.1723	236.1727	1.46	1	607.99	C15H22O2	(M+H)+
237.1803	237.1755	-20.43	1	162.33	C15H22O2	(M+H)+
257.1461	257.1512	19.72	1	304.25	C15H22O2	(M+Na)+
